# Inhibition of cGAS-Mediated Interferon Response Facilitates Transgene Expression

**DOI:** 10.1016/j.isci.2020.101026

**Published:** 2020-03-31

**Authors:** Yajuan Fu, Yijun Fang, Zhang Lin, Lei Yang, Liqun Zheng, Hao Hu, Tingting Yu, Baoting Huang, Suxing Chen, Hanze Wang, Shan Xu, Wei Bao, Qi Chen, Lijun Sun

**Affiliations:** 1 Fujian Key Laboratory of Innate Immune Biology, Biomedical Research Center of South China, College of Life Science, Fujian Normal University Qishan Campus, College Town, Fuzhou, Fujian Province 350117, China; 2 Fujian Normal University Hospital, Fujian Normal University Qishan Campus, College Town, Fuzhou, Fujian Province 350117, China

**Keywords:** Biological Sciences, Molecular Biology, Molecular Mechanism of Gene Regulation

## Abstract

DNA transfection is often the bottleneck of research and gene therapy practices. To explore the mechanism regulating transgene expression, we investigated the role of the cGAS-STING signaling pathway, which induces type-I interferons in response to DNA. We confirmed that deletion of cGAS enhances transgene expression at the protein level by ~2- to 3-fold. This enhancement is inversely correlated with the expression of interferons and interferon stimulated genes (ISGs), which suppress expression of transfected genes at the mRNA level. Mechanistically, DNA transfection activates the cGAS-STING pathway and induces the expression of the OAS family proteins, leading to the activation of RNaseL and degradation of mRNA derived from transgenes. Administration of chemical inhibitors that block cGAS-mediated signaling cascades improves the expression of transgenes by ~1.5- to 3-fold in multiple cell lines and primary cells, including T cells. These data suggest that targeting the cGAS-STING pathway can improve transgene expression, and this strategy may be applied to gene therapy.

## Highlights

• cGAS-STING pathway suppresses transgene expression from DNA vectors
• Interferon-mediated mobilization of OAS-RNaseL system degrades plasmid-derived mRNA
• Inhibitors of cGAS-STING-IFN-ISG axis enhance gene expression in primary cells

## Graphical Abstract

**Figure:**
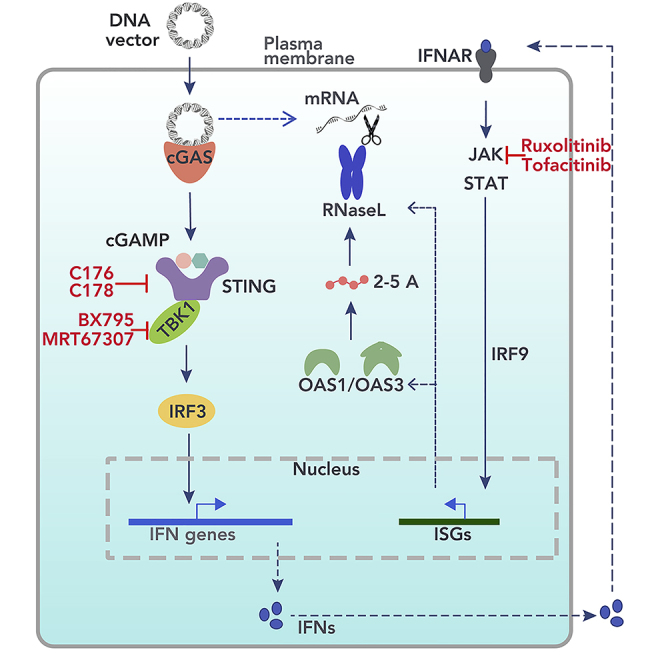

## Introduction

DNA transfection is a common approach to deliver the genes of interest into cells (Kim and Eberwine, 2010) and has been widely used in the study of gene function, producing recombinant proteins including monoclonal antibodies for therapeutic purpose (Jager et al., 2013, Wurm, 2004), and treatment of genetic diseases (Cavazzana-Calvo et al., 2000). Transfection efficiency is critical to achieve a sufficient level of transgene expression in host cells. However, in many cell types, particularly primary cells, transfection efficiency is low (Zhang et al., 2009). A variety of transfection methods have been developed, including DEAE-dextran, branched polyethylenimine (PEI) (Fischer et al., 1999), calcium phosphate coprecipitation, cationic lipid vehicles (such as Lipofectamine), electroporation (Chu et al., 1987), or nucleofection (Kim and Eberwine, 2010, Lai et al., 2003, Niidome and Huang, 2002). All of these methods are focused on improving the delivery of foreign DNA into target cells. However, very little is known regarding what may affect transgene expression within the cell. Previous studies have suggested that foreign DNA including transfected plasmid may stimulate cellular responses to restrain foreign gene expression within the target cells (Bosnjak et al., 2017, Langereis et al., 2015, Sellins et al., 2005); however, the in-depth mechanisms remain unclear.

Cyclic GMP-AMP synthase (cGAS) was previously identified as a major cytoplasmic DNA sensor (Sun et al., 2013). cGAS is a member of the family of nucleotidyl transferases (NTases). Upon binding to DNA, cGAS can use ATP and GTP as substrates to produce 2′,3′-cyclic GMP-AMP (cGAMP) (Gao et al., 2013, Wu et al., 2012), which acts as a second messenger that binds and activates STING (Cai et al., 2014, Chen et al., 2016). Once activated by cGAMP, STING relocates from the endoplasmic reticulum to the Golgi complex and stimulates TBK1 and IKK kinase complexes (Dobbs et al., 2015), resulting in the phosphorylation and activation of transcription factors IRF3 and NF-κB and leading to the production of type I interferons (IFNs) (Barber, 2011, Hiscott, 2007). Through the activation of the Janus kinase (JAK)-signal transducer and activator of transcription (STAT) pathway (Porritt and Hertzog, 2015), type I interferons induce expression of a large array of immune response genes collectively called interferon stimulated genes (ISGs), which establishes an antiviral state to curb replication of invading microbes such as DNA viruses (Katze et al., 2002). Recognition of DNA by cGAS is sequence independent; therefore, transfected DNA is also able to activate the cGAS-STING pathway (Langereis et al., 2015). However, the effect of activation of cGAS-STING pathway on foreign gene expression remains unclear.

Here, we report a role of cGAS-STING and interferon pathways in regulating expression of exogenous genes. We find that IFN and ISGs induced by transfected DNA through the cGAS pathway severely suppress expression levels of transfected genes. Particularly, mobilization of the OAS-RNaseL system is a key factor in restraining transgene expression. Chemical inhibition of cGAS-mediated IFN responses can significantly improve expression levels of transfected genes in multiple cell lines and in primary cells including T cells. Our study uncovers a mechanism through which cytosolic DNA sensing pathway inhibits gene expression from vector DNA and provides a strategic solution to improve transgene expression in primary cells.

## Results

### Knockout of cGAS Increases the Expression Levels of Transfected Genes

**Figure 1:**
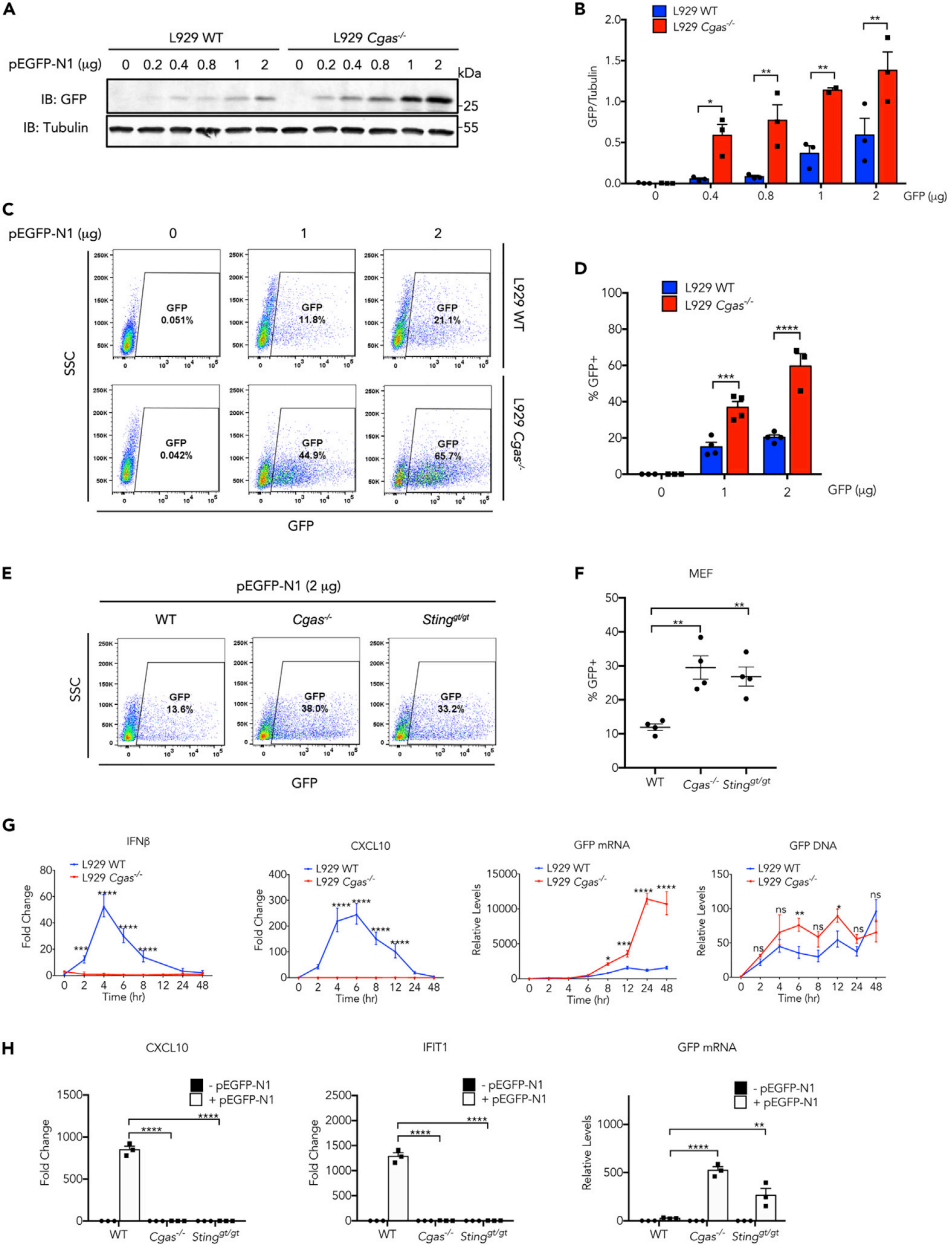
cGAS Inhibits Expression of Transfected Genes (A) pEGFP-N1 plasmid at indicated amount was transfected into wild-type or *Cgas*^*−/−*^ L929 cells. Twenty-four hours later, expression of EGFP protein was detected by western blot analysis. (B) Quantification of intensity of EGFP bands in (A). (C) FACS analysis of GFP-positive cells 24 h after transfection of indicated amount of pEGFP-N1 plasmid into wild-type or *Cgas*^*/−*^ L929 cells. (D) Quantification of GFP-positive cells in (C). (E) FACS analysis of GFP-positive cells 24 h after transfection of pEGFP-N1 plasmid into primary MEFs from wild-type, *Cgas*^*−/−*^, or *Sting*^*gt/gt*^ mice. (F) Quantification of GFP-positive cells in (E). (G) RT-qPCR measurement of RNA levels of IFNβ, CXCL10, and EGFP, and qPCR measurement of levels of EGFP DNA following transfection of pEGFP-N1 into wild-type or *Cgas*^*−/−*^ L929 cells. (H) RT-qPCR quantification of RNA levels of CXCL10, IFIT1, and EGFP following transfection of pEGFP-N1 plasmid into primary MEFs from wild-type, *Cgas*^*−/−*^, or *Sting*^*gt/gt*^ mice. In (B), (D), (G), and (H), data represent mean ± SEM of three independent experiments. ∗p < 0.05, ∗∗p < 0.01, ∗∗∗p < 0.001, and ∗∗∗∗p < 0.0001 by two-way ANOVA with Bonferroni's correction. Ns, not significant (significance level, α = 0.05). In (F), Data represent mean ± SEM of >3 independent experiments. ∗∗p < 0.01 by one-way ANOVA versus WT with Dunnett's correction. See also Figures S1 and S2.

The cGAS-STING signaling pathway is activated by cytosolic DNA including transfected plasmid DNA and leads to production of type I IFNs and ISGs (Cai et al., 2014). To determine whether cGAS-STING-mediated signaling has an effect on the expression of a foreign gene carried by a plasmid, we first generated Cgas-null L929 cells using the CRISPR/Cas9 technology. TA cloning and sequencing data indicated that all three alleles of the cGAS gene were disrupted (Figure S1A); the lack of cGAS protein expression was confirmed by western blot analysis (Figure S1B). Functional assays showed that *Cgas*^*−/−*^ L929 cells were defective in response to DNA transfection but could generate normal levels of IFNβ in response to poly(I:C), a double-stranded RNA analogue that activates RIG-I/Mda5-MAVS pathway (Figure S1C). We then transfected pEGFP-N1 plasmid DNA complexed with Lipofectamine 2000 into wild-type and *Cgas*^-/-^ L929 cells and examined the GFP protein expression by western blot. Knockout of cGAS led to a 2- to 3-fold increase in GFP protein levels 24 h after transfection (Figures 1A and 1B). Consistent with the western blot results, fluorescence microscopy also observed more GFP-positive cells in *Cgas*^*−/−*^ L929 cells than in wild-type (WT) cells after transfection (Figure S1D). The GFP-positive cell population was further quantified by fluorescence-activated cell sorting (FACS) (Figures 1C and 1D); the lack of cGAS in L929 cells led to a >2-fold increase in the population of GFP-positive cells after transfection. A similar effect was also observed in human BJ-5ta cells, wherein the disruption of cGAS gene (Figure S1E) led to an increased percentage of GFP-positive cells in two independent knockout clones after infection with a lentivirus encoding GFP (Figure S1F).

We confirmed the negative effect of DNA sensing pathway on transgene expression in primary cells. We prepared murine embryonic fibroblasts (MEFs) from WT, *Cgas*^*−/−*^, and *Sting*^*gt/gt*^ mice and transfected them with the pEGFP-N1 plasmid. Loss of either cGAS or STING led to >2-fold increase in GFP-positive cells as measured by FACS (Figures 1E and 1F).

To rule out that this phenomenon is due to any peculiarity of the plasmid we were using, we transfected a different plasmid, pGL3-Enhancer, which encodes firefly luciferase, into primary lung fibroblasts from wild-type, *Cgas*^*−/−*^, and *Sting*^*gt/gt*^ mice. Luciferase activity was around 1,000-fold higher in *Cgas*^*−/−*^ and *Sting*^*gt/gt*^ cells than in wild-type cells (Figure S1G, left panel). The more prominent difference between wild-type and deficient cells was likely due to the unusual stability of luciferase protein. Collectively, these results indicate that the cGAS-STING pathway suppresses the expression of foreign genes delivered through plasmid transfection.

To investigate the potential role of RNA sensing pathway in expression of transgenes, we compared the transfection efficiency of GFP in wild-type and *Mavs*^*−/−*^ L929 cells generated using the CRISPR/Cas9 technique (Figures S2A and S2B). *Mavs*^*−/−*^ L929 cells are defective in IFN induction in response to poly(I:C) transfection but have normal IFN and ISG responses to herring testis DNA (HT-DNA) transfection (Figures S2C–S2E). In contrast to *Cgas*^*−/−*^ cells, knocking out MAVS did not affect GFP expression after transfection as indicated by FACS analysis of GFP+ cells (Figure S2F). Transfection of wild-type and *Mavs*^*−/−*^ primary MEFs led to similar levels of GFP expression as demonstrated by western blot (Figure S2G). These results further support the specific role of the cGAS-STING pathway in suppressing the expression of a transgene.

### Plasmid DNA-Induced IFN Response Suppresses Foreign Gene Expression

**Figure 2:**
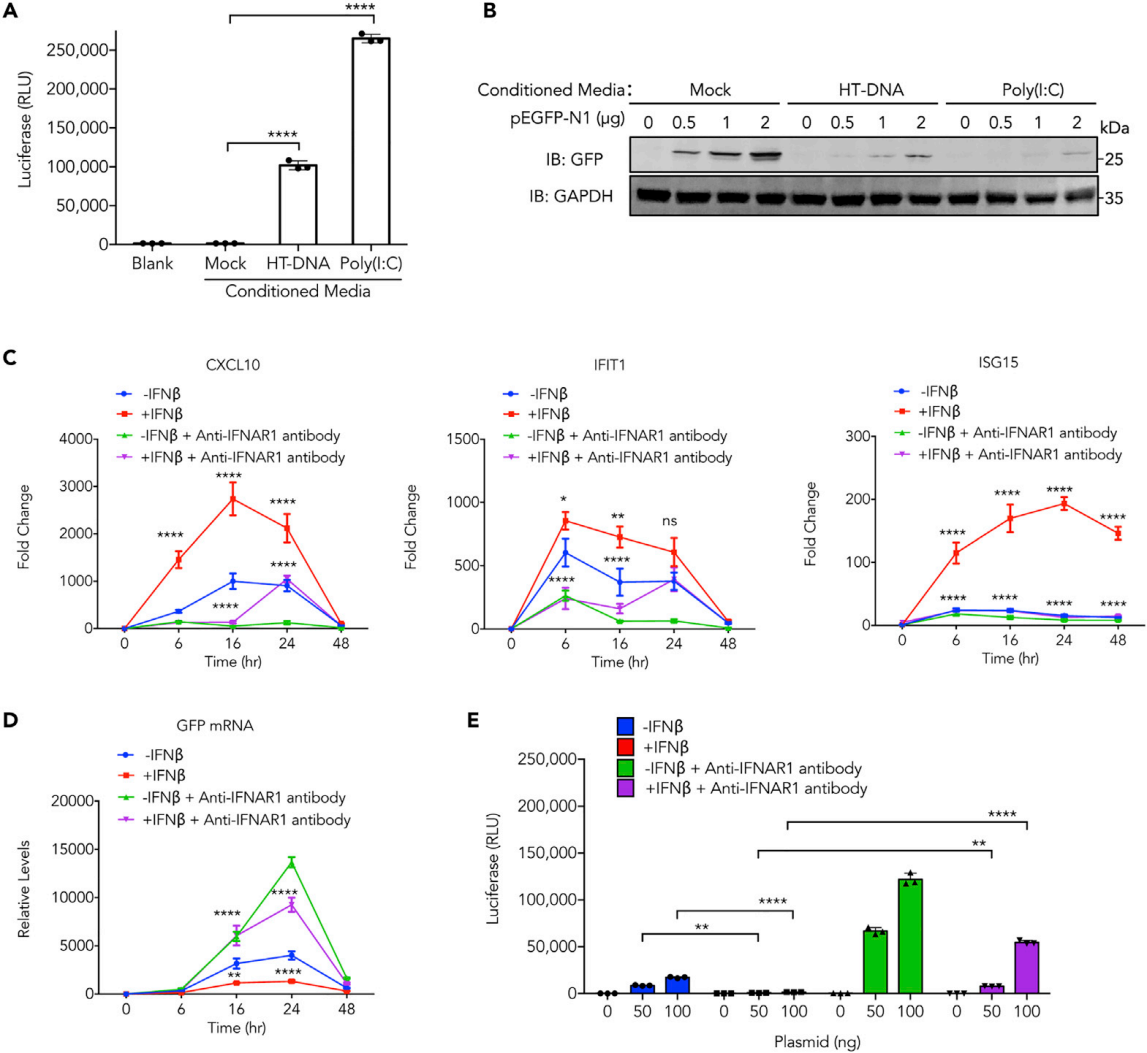
Effects of Interferon on Expression of Exogenous Genes (A) Measurement of interferon activity in conditioned media using Raw-ISG-luc cells. Data represent mean ± SEM. ∗∗∗∗p < 0.0001 by one-way ANOVA versus Mock with Dunnett's correction. (B) L929 cells treated with conditioned media from HT-DNA or poly(I:C) stimulated cells were transfected with indicated amount of pEGFP-N1 plasmid and expression of GFP protein was detected by western blot. (C and D) L929 cells were treated with recombinant IFNβ or anti-IFNAR1 antibody or both, then transfected with pEGFP-N1 plasmid; RNA levels of indicated ISGs (C) and EGFP (D) were measured by RT-qPCR. (E) L929 cells were treated with recombinant IFNβ or anti-IFNAR1 antibody or both, then transfected with pCMV-Renilla plasmid for 20 h, followed by measurement of luciferase activity in cell lysates. In (C)–(E), data represent mean ± SEM of three independent experiments. ∗p < 0.05, ∗∗p < 0.01, ∗∗∗∗p < 0.0001 by two-way ANOVA with Bonferroni's correction, Ns, not significant (significance level, α = 0.05).

To understand the mechanism by which cGAS inhibits foreign gene expression, we compared the expression of IFN and ISGs using RT-qPCR in wild-type and *Cgas*^*−/−*^ L929 cells after transfection of the pEGFP-N1 plasmid. The plasmid transfection led to robust induction of IFNβ and ISGs such as CXCL10 in wild-type but not in *Cgas* ^*−/−*^ L929 cells (Figure 1G, left two panels). The same effect was also observed in primary MEFs, wherein deletion of either *Cgas* or *Sting* abolished plasmid DNA-induced interferon response (Figure 1H). Transfection of another plasmid pGL3-Enhancer encoding luciferase also induced expression of CXCL10 and ISG15 in wild-type but not in *Cgas*^*−/−*^, or *Sting*^*gt/gt*^ lung fibroblasts (Figure S1H). Quantitative PCR indicated that the uptake of plasmid DNA was largely unaffected by cGAS (Figure 1G, right panel); however, the mRNA level of EGFP was elevated in both L929 (Figure 1G, third panel) and primary (Figure 1H right panel) *Cgas*^*−/−*^ cells compared with their wild-type counterparts. The inverse correlation between the IFN response and EGFP mRNA levels suggests interferon and ISGs may have a negative impact on the expression of a foreign gene, possibly through autocrine or paracrine manners. To test this hypothesis, we generated conditioned media from cells that were transfected with HT-DNA or poly(I:C). The presence of IFN activity in these media was confirmed by their stimulatory effect on an ISRE-reporter cell line, Raw-ISG-luc (Figure 2A). We treated L929 cells with these conditioned media and examined their effects on the expression of GFP after plasmid DNA transfection. Pre-treatment with both conditioned media strongly inhibited EGFP protein expression, as indicated by western blot (Figure 2B).

To directly access the effect of type I IFN on foreign gene expression, we pre-treated cells with recombinant IFNβ, anti-IFNAR1 antibody, or the combination of both. As expected, IFNβ treatment led to expression of multiple ISGs, including CXCL10, IFIT1, and ISG15 (Figure 2C). These inductions were suppressed when anti-IFNAR1 antibody, which blocks interferon induced signaling, was added to the media at the same time. Inversely correlated with the expression levels of ISGs, RNA transcript levels from the EGFP plasmid were suppressed by IFNβ treatment, and this suppression was reversed by IFNAR1 neutralizing antibody (Figure 2D). The effect of interferon on transgene expression was demonstrated in a different system, wherein expression levels of *Renilla* luciferase from a transfected plasmid were inhibited by IFNβ and enhanced by IFNAR1 neutralizing antibody (Figure 2E). Taken together, these results support the hypothesis that foreign gene expression is restricted by cGAS-STING pathway that induces IFN responses.

### Small Molecule Inhibitors Targeting cGAS-Induced Signaling Improve Expression of Transfected Genes

Since knockout of the DNA sensor cGAS improved expression of transfected genes, we next asked whether the same improvement could be achieved by blocking cGAS-induced signaling cascade with small molecule inhibitors. We first tested the well-characterized TBK1 inhibitor, BX795, (Clark et al., 2009) in L929 cells. Treatment with BX795 suppressed plasmid DNA-induced IFN and ISG production in a dose-dependent manner (Figure S3A). Remarkably, the EGFP mRNA levels were strongly elevated by increasing concentrations of BX795 (Figure S3B). At the highest concentration used (1 μM), BX795 caused >3-fold increase in the EGFP expression level compared with that in DMSO-treated cells. Consistently, FACS analyses detected increased population of GFP-positive cells as a result of BX795 treatment (Figures S3C and S3D). To rule out the possibility of non-specific effect of BX795, we also tested two other inhibitors against TBK1, Amlexanox (Reilly et al., 2013) and MRT67307 (Clark et al., 2011, Hasan and Yan, 2016, Petherick et al., 2015). Both inhibitors significantly increased the number of GFP-positive cells after transfection with pEGFP-N1 plasmid (Figures S3E–S3H) in L929 cells. These results indicate inhibition of TBK1 as a key step in DNA-induced cGAS signaling cascade can significantly improve expression of transfected genes.

Type I interferons often work in autocrine or paracrine manners. After binding to its receptor, interferon induces the expression of multiple ISGs through activation of Janus kinases (JAKs). Since IFN itself can suppress the expression of a transfected gene, we tested whether inhibition of the IFN pathway with small molecules could also improve transgene expression. We tested two JAK inhibitors, Ruxolitinib and Tofacitinib (Gavegnano et al., 2014, Yoshida et al., 2012), which target JAK1/2 and JAK3, respectively. Both inhibitors at 5 μM concentration successfully suppressed plasmid DNA-induced expression of CXCL10 and ISG15 (Figures S4A and S4B). These inhibitors led to increased levels of EGFP mRNA and protein from plasmid transfection (Figures S4C–S4E). Consistently, flow cytometry analyses detected increased population of GFP-positive cells as a result of treatment with these JAK inhibitors (Figures S4F and S4G). The effect of inhibitors on transgene expression is not limited to one plasmid; mCherry expressed from pcDNA3.1 and pLVX vectors were also enhanced as indicated by FACS analysis (Figures S4H and S4I). These data indicate that inhibition of IFN-induced ISGs can improve the expression of transfected genes.

**Figure 3:**
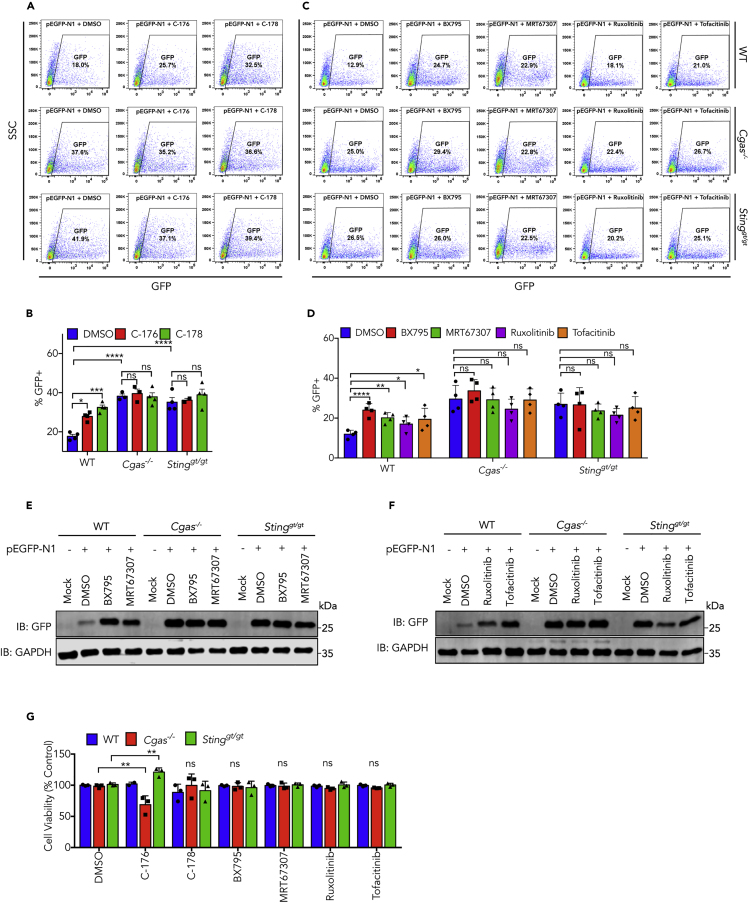
Inhibition of cGAS-Triggered Signaling Increases Transgene Expression in Primary Cells (A) Primary MEFs from wild-type, *Cgas*^*−/−*^, or *Sting*^*gt/gt*^ mice were treated with C-176 and C-178 that covalently inhibit STING, followed by transfection of pEGFP plasmid. GFP-positive cells were analyzed by FACS. Data here represents one of three independent experiments. (B) Quantification of data in (A). (C) Primary MEFs from wild-type, *Cgas*^*−/−*^, or *Sting*^*gt/gt*^ mice were treated with indicated inhibitors targeting TBK1 and JAKs, followed by transfection of pEGFP-N1 plasmid. GFP-positive cells were analyzed by FACS. Data here represents one of >3 independent experiments. (D) Quantification of data in (C). In (B) and (D), data represent mean ± SEM of >3 independent experiments. ∗p < 0.05, ∗∗p < 0.01, ∗∗∗p < 0.001, ∗∗∗∗p < 0.0001 (two-way ANOVA with Tukey's correction). (E and F) The same as (C), except expression of GFP protein was analyzed by western blot. (G) MEFs of indicated genotypes were treated with inhibitors at the following concentrations and cell viability was measured using CCK-8 assay. C-176: 6.25 μM, C-178: 3.125 μM, BX795: 0.5 μM, MRT67307: 0.2 μM, Ruxolitinib: 5 μM, Tofacitinib 5 μM. Data represent mean ± SEM of >3 independent experiments. ∗∗p < 0.01 (two-way ANOVA, with Tukey's correction), Ns, not significant (significance level, α = 0.05). See also Figures S3 and S4.

We took more vigorous approaches by using primary cells to test the role of cGAS and STING in suppression of transgene expression and the effect of chemical inhibitors. In addition to the aforementioned inhibitors, we also included the recently reported STING inhibitors C-176 and C-178, which covalently modify and inhibit STING (Haag et al., 2018). FACS results indicate that pretreatment of MEFs with C-176 and C-178 followed by transfection of pEGFP-N1 plasmid increased the percentage of GFP+ cells (Figures 3A and 3B). Very interestingly, although MEFs from *Cgas*^*−/−*^ or *Sting*^*gt/gt*^ mice exhibited a higher population of GFP+ cells, pretreatment with STING inhibitors did not cause further increase of GFP expression, indicating that the effect of these inhibitors can be exclusively attributed to STING. Similar to the effects in cell lines, TBK1 inhibitors (BX795 and MRT67307) and JAK inhibitors (Ruxolitinib and Tofacitinib) increased GFP+ cell population to various extent in wild-type, but none of them had significant effect in *Cgas*^*−/−*^, or *Sting*^*gt/gt*^ MEFs (Figures 3C and 3D), which already exhibited higher levels of GFP+ cells. Western blot consistently showed that treatment of these cells with inhibitors against TBK1 and JAKs led to higher protein levels of EGFP after transfection in wild-type MEFs. However, in *Cgas*^*−/−*^ or *Sting*^*gt/g*^ cells, these inhibitors had no effect on EGFP protein expression, which are already much higher than wild-type cells (Figures 3E and 3F). Exposure to these compounds had no overt effect on cell viability, except slight changes caused by one of STING inhibitors, C-176 (Figure 3G). Taken together, these data indicate that the cGAS-STING pathway is the major factor in the interferon-related restriction on transgene expression from DNA vectors; inhibition of this pathway and further downstream signaling cascade could overcome the difficulties in transgene expression.

### Degradation of mRNA Derived from Transgenes Is Mediated by the OAS-RNaseL System

Next, we were interested in which factors directly exert the suppressing effect on transgenes. It has been reported that protein kinase R (PKR) can shut down translation by phosphorylating eukaryotic translation initiation factor eIF2α (Garcia et al., 2006, Ilan et al., 2017). We generated *Pkr*^*−/−*^ L929 cells using the CRISPR/Cas9 technology (Figure S5A) and compared the transfection efficiency in wild-type, *Pkr*^*−/−*^, and *Cgas*^*−/−*^ L929 cells. In contrast to the knockout of *Cgas* gene, which increased GFP+ cells by 2- to 3-fold, knockout of *Pkr* had no effect (Figure S5B). We also compared the effect of PKR inhibitor (CAS 608512-97-6) with that of TBK1 inhibitor BX795 in the same transfection experiment. Unlike BX795, the PKR inhibitor did not increase the percentage of GFP+ cells (Figure S5C). These results ruled out PKR as a factor responsible for suppression of foreign gene expression from transfected plasmids.

**Figure 4:**
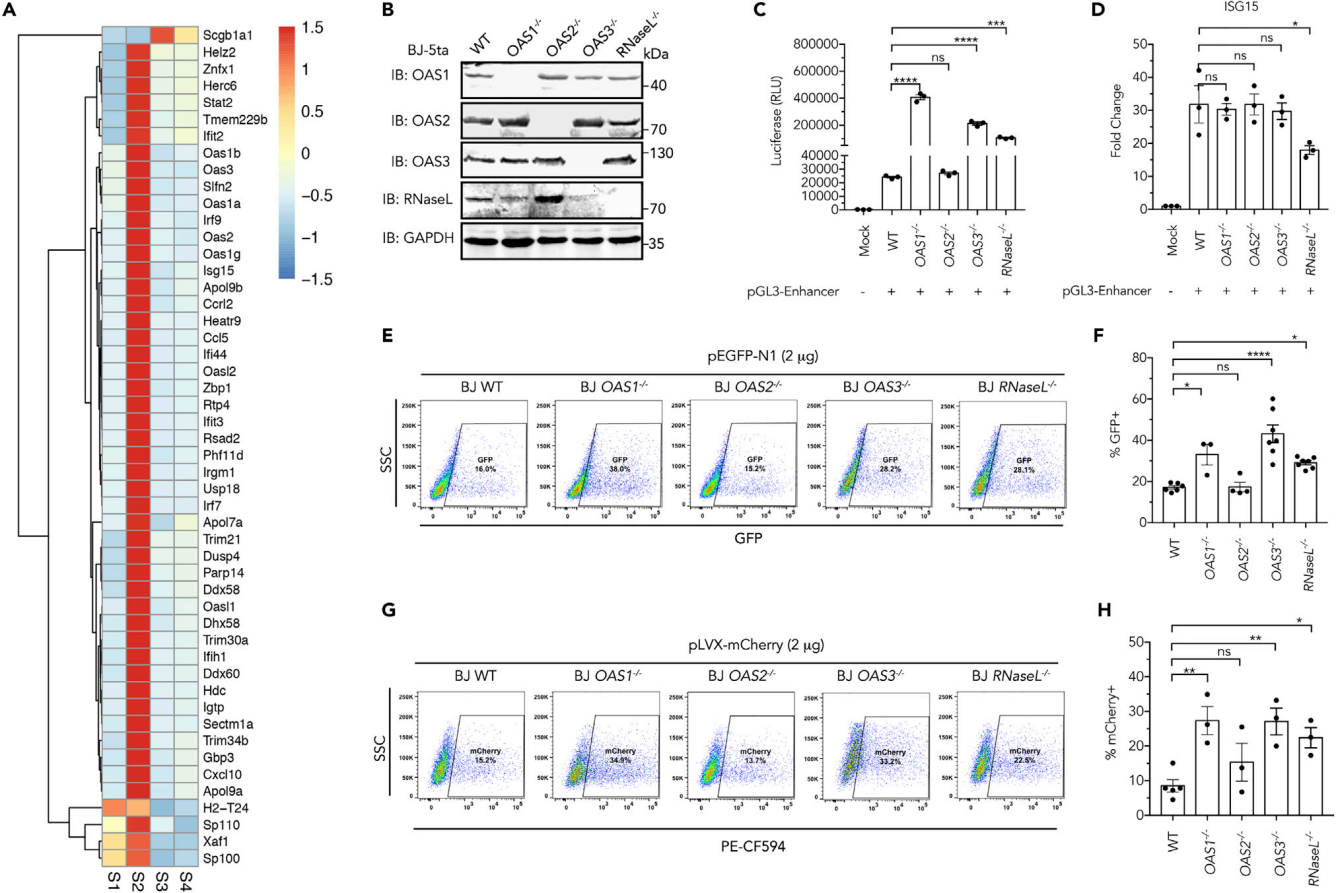
The OAS-RNaseL System Suppresses Transgene Expression (A) Heatmaps of RNA-seq analyses. S1, L929 cells mock-treated; S2, L929 cells transfected with pEGFP-N1 plasmid; S3, L929 treated with 0.5 μM BX795; S4, L929 treated with 0.5 μM BX795 and transfected with pEGFP-N1 plasmid. All samples were collected after 6 h. Color gradient indicates fold changes in log2 scale. Genes in sample S2 exhibiting statistically significant (p < 0.05), two-fold or greater increases over S1, S3, S4 are shown. (B) Western blot of indicated proteins in wild-type, *OAS1*^*−/−*^, *OAS2*^*−/−*^, *OAS3*^*−/−*^, or *RNaseL*^*−/−*^ (CRISPR-Cas9) BJ-5ta cells. (C) Luciferase activity in extracts from indicated wild-type or CRISPR-Cas9 knockout cell lines 24 h after transfection of pGL3-Enhancer plasmids. (D) Expression levels of ISG15 as measured by RT-qPCR 8 h after transfection of indicate BJ-5ta cells with pGL3-Enhancer plasmid. (E−H) BJ-5ta cells of indicated genotypes were transfected with pEGFP-N1 plasmid (E) or pLVX-mCherry (G) using lipofectamine method; 24 h later, fluorescence-positive cells were analyzed by FACS. (F) and (H) are quantification of GFP- or mCherry-positive cells in (E) and (G), respectively. In (C), (D, (F), and (H), data represent mean ± SEM of ≥3 independent experiments. ∗p < 0.05, ∗∗p < 0.01, ∗∗∗p < 0.001, ∗∗∗∗p < 0.0001 (one-way ANOVA, with Dunnett's correction). Ns, not significant (significance level, α = 0.05). See also Figures S5 and S6.

We performed gene expression profile analyses using the RNA-seq technology. L929 cells were pretreated with TBK1 inhibitor BX795 or mock treated with DMSO, then transfected with pEGFP-N1 plasmid. We confirmed the increased expression of EGFP in BX795-treated cells, as well as the induction of IFNβ, CXCL10, and ISG15 by transfection and their inhibition by BX795 (Figure S6A). RNA-seq data indicate that transfection of plasmid led to upregulation of a large number of genes, which were suppressed by BX795 (Figure 4A, GEO: GSE144913). Gene categories related to “response to virus,” which include many ISGs (Schneider et al., 2014), are the most enriched genes according to gene ontology analysis (Figure S6B). We hypothesized that some of the ISGs may directly suppress expression of transfected genes.

Since suppression of foreign gene expression occurred at the mRNA level (Figure 1G), we turned our attention to ISGs that may affect RNA turnover. The 2′,5′-oligoadenylate synthetase (OAS)-RNaseL pathway plays an important role in antiviral responses (Silverman, 2007). IFNs upregulate OAS proteins, which on encounter with viral double-stranded RNA synthesize 2′,5′-oligoadenylate (2-5OA). As a ligand, 2-5OA triggers enzymatic activity of RNaseL, which degrades viral and cellular RNA. In our RNA-seq results, we found that all three OAS isoforms (OAS1–3) were upregulated after transfection of the EGFP plasmid, and their upregulation was suppressed by BX795; this result was also confirmed by RT-qPCR analyses (Figure S6C). To investigate the potential role of OAS family proteins and RNaseL in suppressing foreign gene expression, we deleted the individual OAS genes as well as RNaseL gene in BJ-5ta cells using the CRISPR-Cas9 technology (Figure S6D). Western blots using the antibodies against each individual OAS isoforms and RNaseL confirmed knockout (Figure 4B). When we transfected these cells with the pGL3 plasmid that carries a luciferase gene, *OAS1*^*−/−*^*, OAS3*^*−/−*^*,* and *RNaseL*^*−/−*^ cells exhibited 5- to 15-fold higher luciferase activity compared with wild-type cells (Figure 4C). Surprisingly, knockout of OAS2 did not lead to higher expression of luciferase. As predicted, transfection of plasmid DNA caused induction of ISG15 in all cells (Figure 4D). When these cells were transfected with two other plasmids, pEGFP-N1 or pLVX-mCherry, the proportion of fluorescence-positive cells was enhanced ~1.5- to 3-fold in *OAS1*^*−/−*^*, OAS3*^*−/−*^*,* and *RNaseL*^*−/−*^ but not in *OAS2*^*−/−*^ cells (Figures 4E–4H). These results suggest that activation of OAS1, OAS3, and RNaseL leads to degradation of mRNA and suppression of transgene expression.

**Figure 5:**
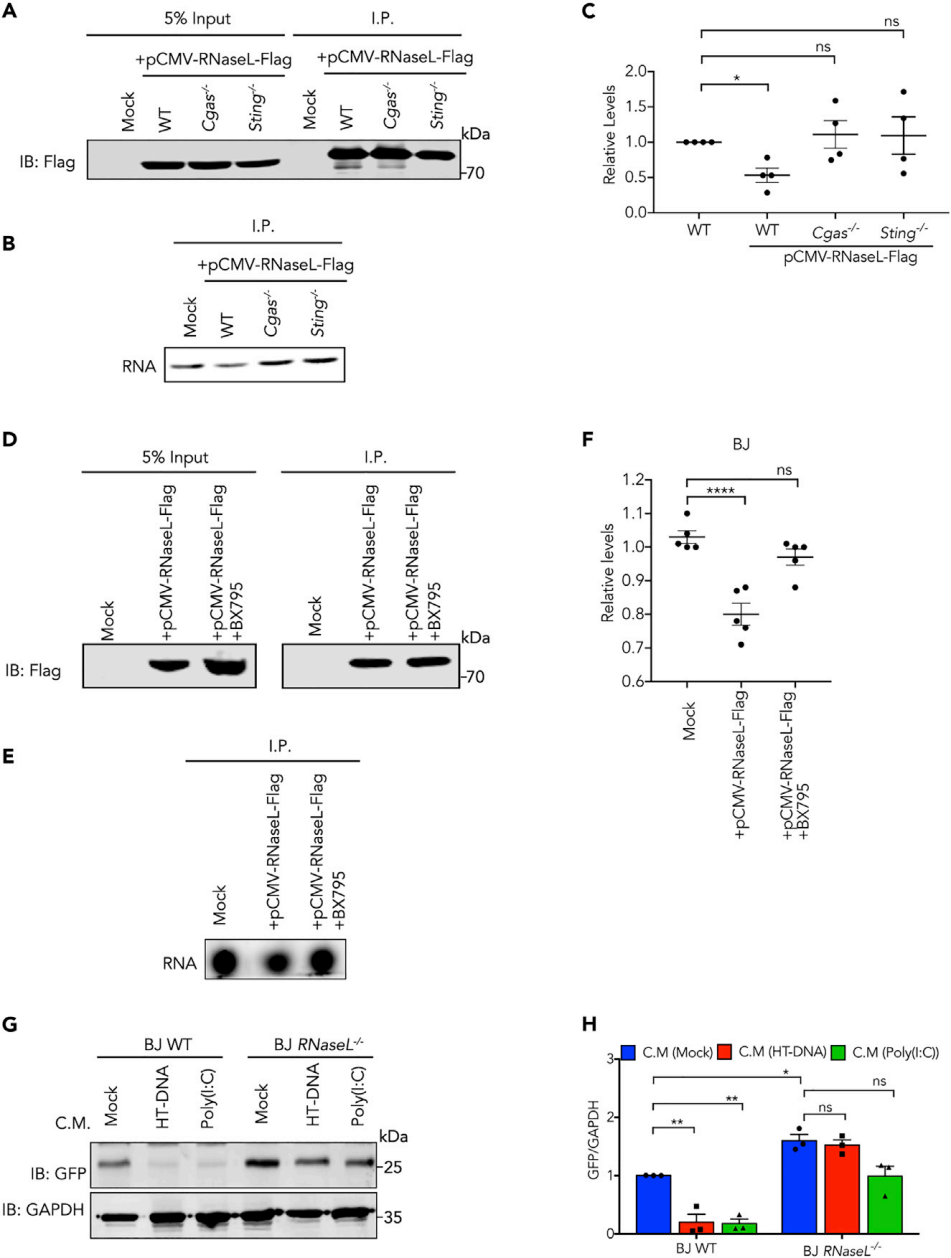
DNA Transfection Induces RNaseL Activity through cGAS-STING and Interferon Pathways (A) L929 cells of indicated genotypes were transfected with a plasmid expressing Flag tagged RNaseL. Lysates containing comparable total amount of RNAseL-Flag protein were subjected to immunoprecipitation (IP) with Flag(M2) beads. Western blot showing levels of RNaseL-Flag in 5% input and IP. (B) RNaseL activities in IP products in (A) were measured by incubation with a fluorescent RNA oligonucleotide and gel electrophoresis. (C) Quantification of band intensity in (B). (D) BJ-5ta cells were treated with BX795 or DMSO and transfected with a plasmid expressing Flag tagged RNaseL, which was immunoprecipitated (IP) with Flag(M2) beads. Western blot showing levels of RNaseL-Flag in input and IP. (E) RNaseL activities in IP products in (D) were measured by incubation with a fluorescent RNA oligonucleotide and gel electrophoresis. (F) Quantification of band intensity in (E). In (C) and (F), data represent mean ± SEM of >3 independent experiments. ∗p < 0.05, ∗∗∗∗p < 0.0001 (one-way ANOVA versus WT or Mock, with Dunnett's correction). Ns, not significant. (G) Wild-type and *RNaseL*^*−/−*^ BJ-5ta cells were treated with conditioned media from cells exposed to HT-DNA or Poly(I:C), then transfected with pEGFP-N1 plasmid for 24 h; levels of GFP protein was analyzed by western blot. (H) Quantification of GFP bands in (G). Data represent mean ± SEM of three independent experiments. ∗p < 0.05, ∗∗p < 0.01 (two-way ANOVA, Tukey's correction). Ns, not significant.

To directly detect RNaseL activity in cells following plasmid DNA delivery, we transfected a plasmid encoding RNaseL with a FLAG tag at C-terminal (pCMV-RNaseL-Flag) into wild-type, *Cgas*^*−/−*^, and *Sting*^*−/−*^ L929 cells. To measure RNaseL activation, we immunoprecipitated (IP) RNaseL-Flag using anti-Flag beads from cell lysates; the input was adjusted so that the starting materials contained similar amounts of the RNaseL-Flag protein (Figure 5A). We then incubated the IP products with an RNA probe that served as the substrate for RNaseL. Gel electrophoresis showed the intensity of the RNA band was reduced by IP product from wild-type but not Cgas^−/-^ or *Sting*^*−/−*^ cells (Figures 5B and 5C), indicating RNaseL activity was indeed triggered by DNA transfection in a cGAS-STING-dependent manner. To test the effect of inhibitors of cGAS-induced signaling cascade on RNaseL activation, BJ-5ta cells were transfected with pCMV-RNaseL-Flag in the presence of TBK1 inhibitor BX795 or DMSO; anti-Flag IP products (Figure 5D) were tested for their RNaseL activity. As shown in Figures 5E and 5F, plasmid DNA-induced RNaseL activity was abolished in BX795-treated cells. These results again confirm that the cGAS-STING pathway restricts transgene expression through activation of RNaseL. To evaluate the degree of contribution of RNaseL to this effect, we treated wild-type or *RNaseL*^*−/−*^ BJ-5ta cells with conditioned media from cells exposed to HT-DNA, poly(I:C), or mock treated, and transfected these cells with pEGFP-N1 plasmid. Consistent with results in Figure 2B, pretreatment of cells with conditioned media significantly reduced EGFP expression from plasmid as shown by western blot (Figures 5G and 5H); however, the effect of conditioned media was diminished in *RNaseL*^*−/−*^ BJ-5ta cells. These results support the major role of RNaseL in suppressing transgene expression.

### Chemical Inhibitors Improve Transfection in Primary T Cells

**Figure 6:**
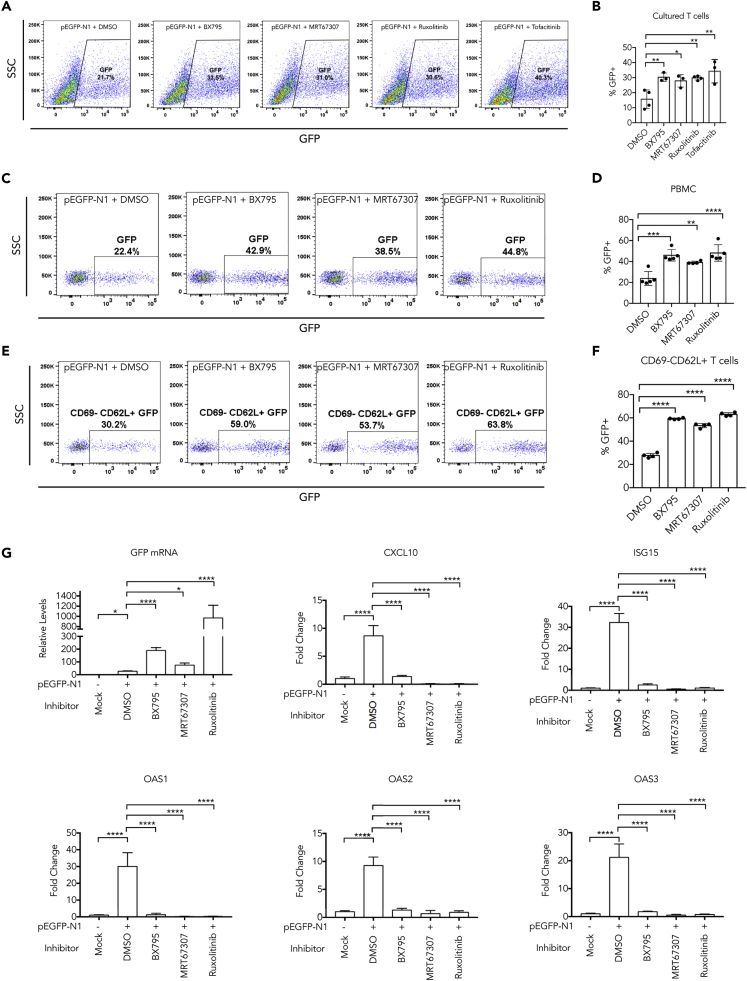
Inhibition of DNA-Induced Signaling Pathway Enhances Transgene Expression in T Cells (A) T cells expanded from human PBMC using CD3 and IL2 (see Transparent Methods) were treated with indicated inhibitors or DMSO and electroporated with pEGFP-N1 plasmid. Twenty-four hours later, cells were analyzed for GFP expression using FACS. (B) Quantification and statistics of data in (A). (C) Human PBMCs isolated using Ficoll gradient were transfected with pEGFP-N1 plasmid for 24 h; lymphocytes population from FSC/SSC gating was analyzed for EGFP-positive cells. (D) Quantification and statistics of data in (C). (E) The same as in (C), except PBMCs were further gated on CD3+, CD69-, CD62L+, and GFP to analyze EGFP expression in resting T cells. (F) Quantification and statistics of data in (E). (G) Quantification of mRNA levels of GFP or indicated ISGs by RT-PCR after proliferated human T cells (as in A) were treated with DMSO or inhibitors and transfected with pEGFP-N1 plasmid. Data represent mean ± SEM of at least three independent experiments. ∗p < 0.05, ∗∗p < 0.01, ∗∗∗p < 0.001, and ∗∗∗∗p < 0.0001 by one-way ANOVA versus DMSO, with Dunnett's correction. See also Figure S7.

Transfection of T cells remains a great challenge in research and gene therapy practices. We therefore examined whether chemical inhibitors that target DNA-induced IFN response can improve gene expression from plasmid transfection in T cells. We first isolated human peripheral blood mononuclear cells (PBMCs) from healthy donors using Ficoll density centrifugation, then stimulated them with IL-2/CD3/CD28 for 10 days to obtain sufficient cells. Flow cytometry confirmed >95% of cells were CD3+ T cells. We then treated these cells with inhibitors or DMSO and delivered EGFP plasmid through electroporation. Inhibitors targeting TBK1 (BX795 and MRT67307) and JAKs (Ruxolitinib and Tofacitinib) led to enhanced (~2-fold) expression of GFP in these T cells 24 h after transfection as quantified by FACS (Figures 6A and 6B). Exposure to these inhibitors did not alleviate cell death commonly associated with electroporation, but neither did they have adverse effect on cell growth up to 18 days after electroporation (Figure S7). We also tested these inhibitors in PBMC and resting T cells. BX795 and Ruxolitinib led to a nearly 2-fold increase of GFP+ cells in both bulk PBMCs (Figures 6C and 6D) and CD69-CD62L+ resting T cells (Figures 6E and 6F). MRT67307 exhibited less but still significant improvement of transfection in these cells. Enhancement of EGFP gene expression in proliferated T cells is more prominent at mRNA levels (Figure 6G, first panel). Consistent with previous findings, transfection of plasmid DNA also led to upregulation of ISGs including OAS1-3 in T cells, which are suppressed by inhibitors (Figure 6G). These results provided evidence that simple administration of small molecule inhibitors can overcome the difficulties in T cell transfection caused by activation of cGAS-STING pathway.

## Discussion

DNA transfection is commonly used to express genes of interest in host cells (Kim and Eberwine, 2010). In many cases, primary cells including T cells remain difficult to transfect, which impedes the application of DNA transfection in gene and cell therapies, such as chimeric antigen receptor (CAR) T cell (CAR-T cells) immunotherapy. In the present study, we investigated the underlying mechanism that controls transgene gene expression by exploring the role of cGAS-STING-mediated DNA sensing pathway. We found plasmid DNA, through cGAS-STING pathway, induced interferon responses. Particularly, mobilization of OAS-RNaseL anti-viral system is the predominant force that causes degradation of mRNA and suppression of transgene expression. Chemical inhibitors that disrupt the signaling cascades originated from DNA-sensing pathway effectively lifted cellular suppression on transgene expression in multiple cell lines and primary cells including T cells.

Under certain conditions, cytosolic DNA can be indirectly sensed by the RIG-I/MAVS pathway. RNA polymerase III can generate transcripts from AT-rich region of the foreign DNA. These transcripts bear 3′-tri-phosphate and are legitimate ligands for RIG-I and can potentially induce IFN response through MAVS (Chiu et al., 2009). However, the plasmid used in this study induced IFN response independent of MAVS. No difference of expression levels of transgene was observed between wild-type and *Mavs*^*−/−*^ cells, suggesting RIG-I/MAVS pathway was not activated by the plasmid. It is possible that other plasmids with particular sequence may be able to trigger interferon response through the RIG-I/MAVS pathway; however, its negative effect on transfection should also be overcome using inhibitors against TBK1 and JAKs.

In addition to IFN response, activation of cGAS-STING pathway by DNA also triggers autophagy through mechanisms that are independent of the TBK1/IRF3 axis (Gui et al., 2019, Liu et al., 2019). We did observe conversion of LC3 into the lipidated form (LC3-II), a hallmark of autophagy, following transfection of plasmids (Figure S8). However, the levels of plasmid DNA inside the cells were not changed when cGAS was knocked out (Figure 1G). We speculated plasmid DNA-triggered autophagy was much weaker than that induced by ISD transfection or permeabilization of cGAMP; therefore, its effect on transgene expression is relatively minor. It is also possible the majority of transfected DNA is hidden in the nucleus; therefore, it is not affected by autophagy. It remains interesting to further explore the role of autophagy in transgene expression.

OAS proteins are among the ISGs that are upregulated after virus infection. Double-stranded RNA (dsRNA) of the viral origin binds and activates OAS1-3, leading to production of 2′-5′-phosphodiester-linked oligoadenylates (2-5OAs), which in turn triggers enzymatic activity of RNaseL. In this study, we have shown evidence that plasmid transfection can lead to RNaseL activation through OAS1 and 3, but not OAS2. These results raised questions regarding the source of dsRNA. One possibility is that certain features of mRNAs transcribed from the plasmid can serve as OAS ligands. The plasmids-originated mRNA species are transcribed by RNA polymerase II and are 5′-capped; this prevents them from activating RIG-I, which requires 5′-triphosphate (Hornung et al., 2006). Using the RNAfold webserver (http://rna.tbi.univie.ac.at//cgi-bin/RNAWebSuite/RNAfold.cgi), we found EGFP mRNA can form secondary structures with a number of stem loops, most of which are too short to activate Mda5 but long enough to activate OAS proteins, such as OAS1, which requires only 17 base pairs (Donovan et al., 2013). The stem loops derived from the EGFR mRNA is reminiscent of the same structures in the genome of West Niles virus, which are directly recognized by OAS1 (Deo et al., 2014). Activation of OAS enzymes may also be favored by the combination of upregulation of OAS proteins and high levels of transcripts as a result of transient transfection. Differential activation of OAS isoforms can be caused by at least two factors. One is that the binding affinity to dsRNA can be very different between OAS isoforms (Ibsen et al., 2014); the other is that certain features on dsRNA can still have an impact on its ability to activate OAS (Schwartz and Conn, 2019). It would be interesting to identify the exact RNA structures that trigger OAS activation following plasmid transfection.

T cells belong to adaptive immune cells and are not typically associated with type I interferon response. However, recent studies have shown that STING is expressed at high levels in most T cells (Larkin et al., 2017) and cGAS is expressed in memory T cells. Although cGAS protein is undetectable in naive T cells using western blot (Cerboni et al., 2017), this does not necessarily rule out its expression at a low level, which is often sufficient for signaling. Moreover, activation of T cells with IL2 and CD3 dramatically increased cGAS expression. Consistent with these reports, we also observed ISG induction in T cells upon transfection of plasmid DNA, confirming an intact cGAS-STING pathway in T cells. More importantly, ISG induction can be entirely blocked by simple addition of small molecule inhibitors of TBK1 and JAKs, which led to significant improvement of transfection efficiency. An important step in CAR-T therapy is expansion of isolated T cells, which will enable cGAS-STING pathway that may impede subsequent delivery of CAR gene. We predict that inhibition of innate immune response will significantly benefit CAR-T engineering and other gene therapy processes.

### Limitation of the Study

Although we have demonstrated the role of cGAS-STING pathway in suppression of transgene expression in primary MEFs, we were unable to demonstrate this directly using primary Cgas^−/−^ and Sting^−/−^ T cells owing to technical limitations.

## Methods

All methods can be found in the accompanying Transparent Methods supplemental file.

## Data and Code Availability

RNA sequencing data were deposited in NCBI GEO database under the accession number GSE144913.

## Supplemental Information

Document S1. Transparent Methods and Figures S1–S8
