# Supplementary material for: Inhibition of cGAS-Mediated Interferon Response Facilitates Transgene Expression
Source: iScience. 2020 Mar 31;23(4):101026. doi: 10.1016/j.isci.2020.101026 (PMC7155207; doi:10.1016/j.isci.2020.101026)
Supplement: Document S1. Transparent Methods and Figures S1–S8 [file mmc1.pdf]

iScience, Volume *23*

Supplemental Information

Inhibition of cGAS-Mediated Interferon Response Facilitates Transgene Expression

Yajuan Fu, Yijun Fang, Zhang Lin, Lei Yang, Liqun Zheng, Hao Hu, Tingting Yu, Baoting Huang, Suxing Chen, Hanze Wang, Shan Xu, Wei Bao, Qi Chen, and Lijun Sun

Figure S1

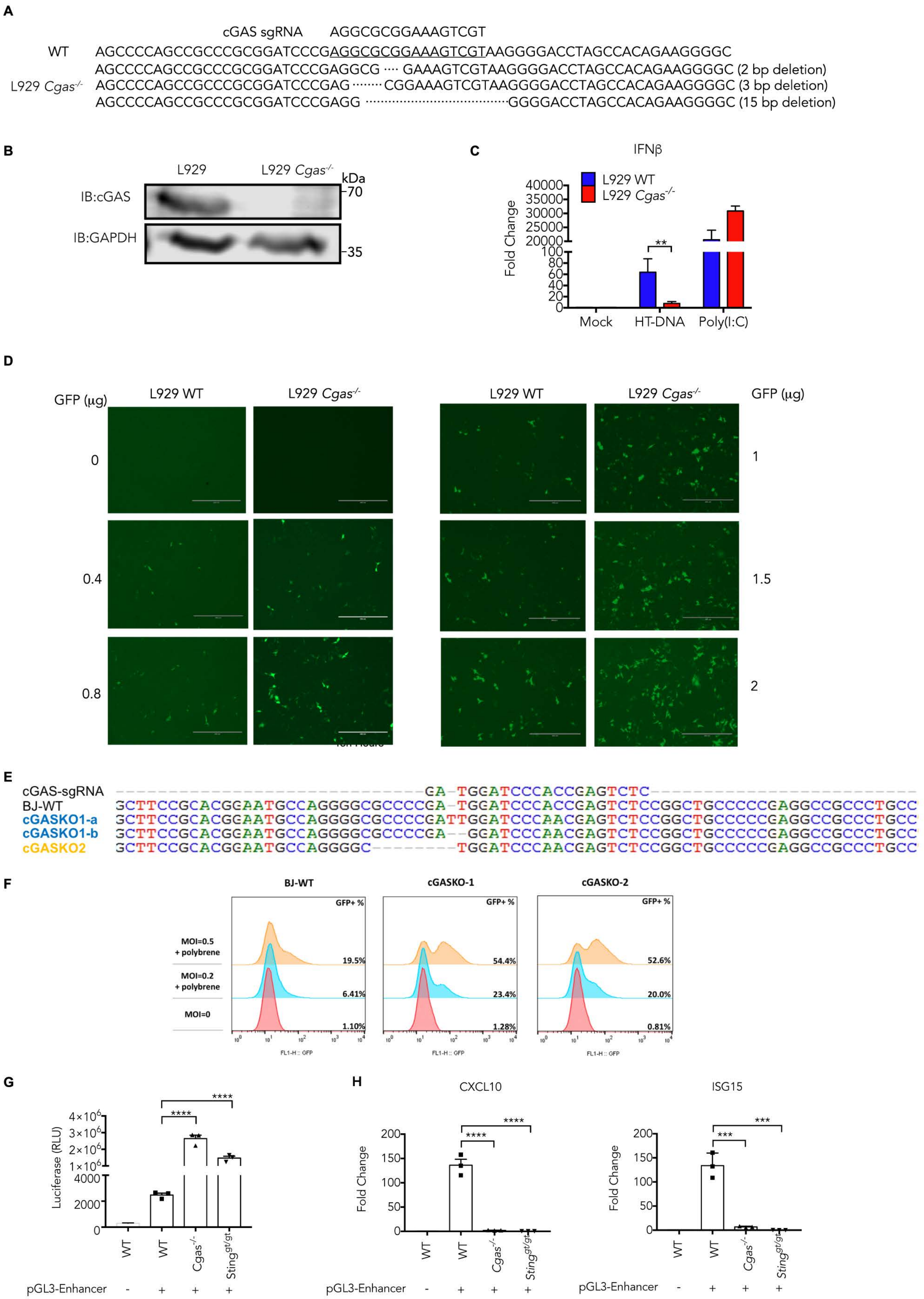
A
cGAS sgRNA AGGCGCGGAAAGTCGT
WT AGCCCCAGCCGCCCGCGGATCCCGAGGCGCGGAAAGTCGTAAGGGGACCTAGCCACAGAAGGGGC
AGCCCCAGCCGCCCGCGGATCCCGAGGCG ···· GAAAGTCGTAAGGGGACCTAGCCACAGAAGGGGC (2 bp deletion)
L929 Cgas-/- AGCCCCAGCCGCCCGCGGATCCCGAG ······· CGGAAAGTCGTAAGGGGACCTAGCCACAGAAGGGGC (3 bp deletion)
AGCCCCAGCCGCCCGCGGATCCCGAGG ································ GGGGACCTAGCCACAGAAGGGGC (15 bp deletion)
B
L929
L929 Cgas-/-
kDa
70
35
IB:cGAS
IB:GAPDH
C
IFNβ
L929 WT
L929 Cgas-/-
Fold Change
40000
35000
30000
25000
20000
100
80
60
40
20
0
**
Mock
HT-DNA
Poly(I:C)
D
GFP (μg)
L929 WT
L929 Cgas-/-
0
0.4
0.8
1
1.5
2
E
cGAS-sgRNA GA TGGATCCCACCGAGTCTC
BJ-WT GCTTCCGCACGGAATGCCAGGGGCGCCCCGA-TGGATCCCACCGAGTCTCCGGCTGCCCCCGAGGCCGCCCTGCC
cGASKO1-a GCTTCCGCACGGAATGCCAGGGGCGCCCCGATTGGATCCCAACGAGTCTCCGGCTGCCCCCGAGGCCGCCCTGCC
cGASKO1-b GCTTCCGCACGGAATGCCAGGGGCGCCCCGA--GGATCCCAACGAGTCTCCGGCTGCCCCCGAGGCCGCCCTGCC
cGASKO2 GCTTCCGCACGGAATGCCAGGGGC-------TGGATCCCAACGAGTCTCCGGCTGCCCCCGAGGCCGCCCTGCC
F
BJ-WT
cGASKO-1
cGASKO-2
GFP+ %
MOI=0.5 + polybrene
MOI=0.2 + polybrene
MOI=0
19.5%
6.41%
1.10%
54.4%
23.4%
1.28%
52.6%
20.0%
0.81%
FL1-H :: GFP
G
Luciferase (RLU)
4×10^6
3×10^6
2×10^6
1×10^6
4000
2000
0
****
****
WT
WT
Cgas-/-
Sting gt/gt
pGL3-Enhancer - + + +
H
CXCL10
ISG15
Fold Change
200
150
100
50
0
****
****
***
***
WT
WT
Cgas-/-
Sting gt/gt
pGL3-Enhancer - + + +

Figure S1. Related to Figure 1. **Knockout of cGAS enhances transgene expression**. **A)** Sequence analysis of *Cgas*$^{-/-}$ L929 cells. Three alleles of *Cgas* gene in L929 each has a frame-shift deletion. **B**) Western blot for cGAS and GAPDH protein levels in WT or *Cgas*$^{-/-}$ L929 cells. **C**) Wild-type or *Cgas*$^{-/-}$ L929 cells were transfected with HT-DNA or poly(I:C). Induction of IFNβ RNA was quantified by RT-qPCR. Data represent mean ± SEM of 3 independent experiments. **$p<0.01$ (two-way ANOVA, Bonferroni's correction). **D**) Fluorescence microscopy of GFP expression 24 hours after transfection of wild-type and *cGAS*$^{-/-}$ L929 cells with increasing amount of pEGFP-N1 plasmid. **E)** Sequence analysis of *cGAS*$^{-/-}$ BJ-5ta (CRISPR) cells showing disruption of alleles. **F)** FACS analysis of GFP expression in wild-type or *cGAS*$^{-/-}$ BJ-5ta cells 48 hours after infection with lentivirus carrying GFP gene. **G and H)** Expression levels of luciferase (G), CXCL10 and Isg15 (H) following transfection of pGL3-Enhancer into lung fibroblasts from wild-type, *Cgas*$^{-/-}$, or *Sting*$^{gt/gt}$ mice. Data represent mean ± SEM of 3 independent experiments. ***$p<0.001$, *and* ****$p<0.0001$ (one-way ANOVA vs WT+ pGL3-Enhancer, with Dunnett's correction).

Figure S2

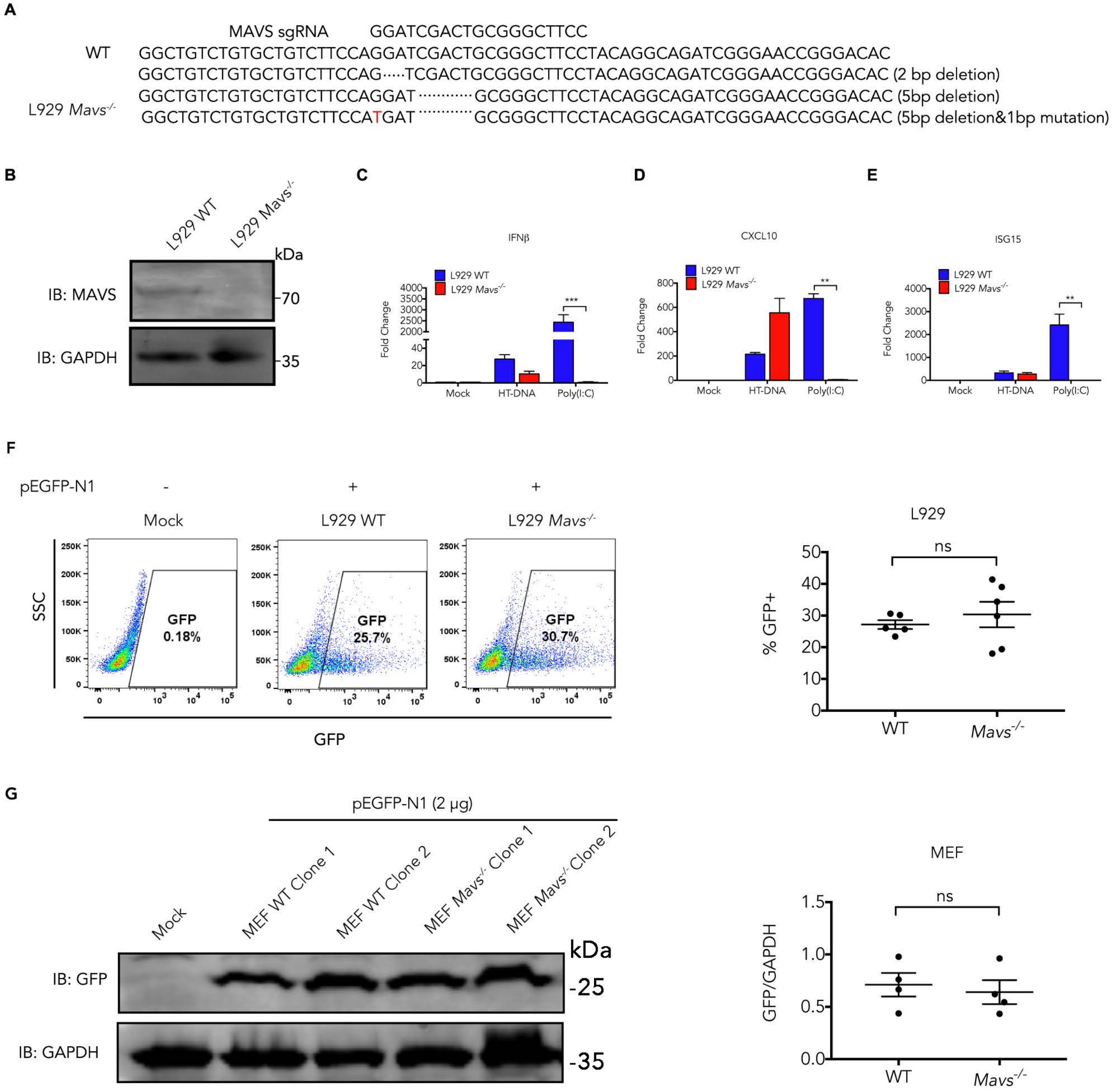

A
MAVS sgRNA GGATCGACTGCGGGCTTCC
WT GGCTGTCTGTGCTGTCTTCCAGGATCGACTGCGGGCTTCCTACAGGCAGATCGGGAACCGGGACAC
GGCTGTCTGTGCTGTCTTCCAG·····TCGACTGCGGGCTTCCTACAGGCAGATCGGGAACCGGGACAC (2 bp deletion)
GGCTGTCTGTGCTGTCTTCCAGGAT···········GCGGGCTTCCTACAGGCAGATCGGGAACCGGGACAC (5bp deletion)
L929 Mavs-/-
GGCTGTCTGTGCTGTCTTCCATGAT···········GCGGGCTTCCTACAGGCAGATCGGGAACCGGGACAC (5bp deletion&1bp mutation)
B
L929 WT
L929 Mavs-/-
kDa
IB: MAVS
-70
IB: GAPDH
-35
C
IFNβ
L929 WT
L929 Mavs-/-
Fold Change
Mock
HT-DNA
Poly(I:C)
D
CXCL10
E
ISG15
F
pEGFP-N1
Mock
L929 WT
GFP 0.18%
GFP 25.7%
GFP 30.7%
SSC
GFP
L929
ns
% GFP+
WT
Mavs-/-
G
pEGFP-N1 (2 μg)
Mock
MEF WT Clone 1
MEF WT Clone 2
MEF Mavs-/- Clone 1
MEF Mavs-/- Clone 2
IB: GFP
-25
-35
MEF
GFP/GAPDH

Figure S2. Related to Figure 1. **MAVS has no effect on expression of transfected genes**. **A**) DNA sequence analysis of *Mavs*$^{-/-}$ L929 cells. **B**) Western blot of MAVS protein in wild-type or *Mavs*$^{-/-}$ L929 cells. **C-E**) Wild-type or *Mavs*$^{-/-}$ L929 cells were stimulated with HT-DNA or poly(I:C), expression of IFNβ, CXCL10, and ISG15 were measured using RT-qPCR. Data represent mean ± SEM. *$^{**}p<0.01$, $^{***}p<0.001$* (two-way ANOVA, Bonferroni's correction). **F**) Wild-type or *MAVS*$^{-/-}$ L929 cells were transfected with pEGFP-N1 plasmid; 48 hours later, GFP positive cells were analyzed using FACS. Right panel shows summary of multiple independent experiments (n=5). Ns: not significant by Student's *t*-test. G) pEGFP-N1 was transfected into primary MEFs from wild-type and *Mavs*$^{-/-}$ mice for 24 hours; EGFP protein was detected by Western Blot. Right panel shows quantification of GFP bands intensity from 4 independent experiments. Ns: not significant by Student's *t*-test.

Figure S3

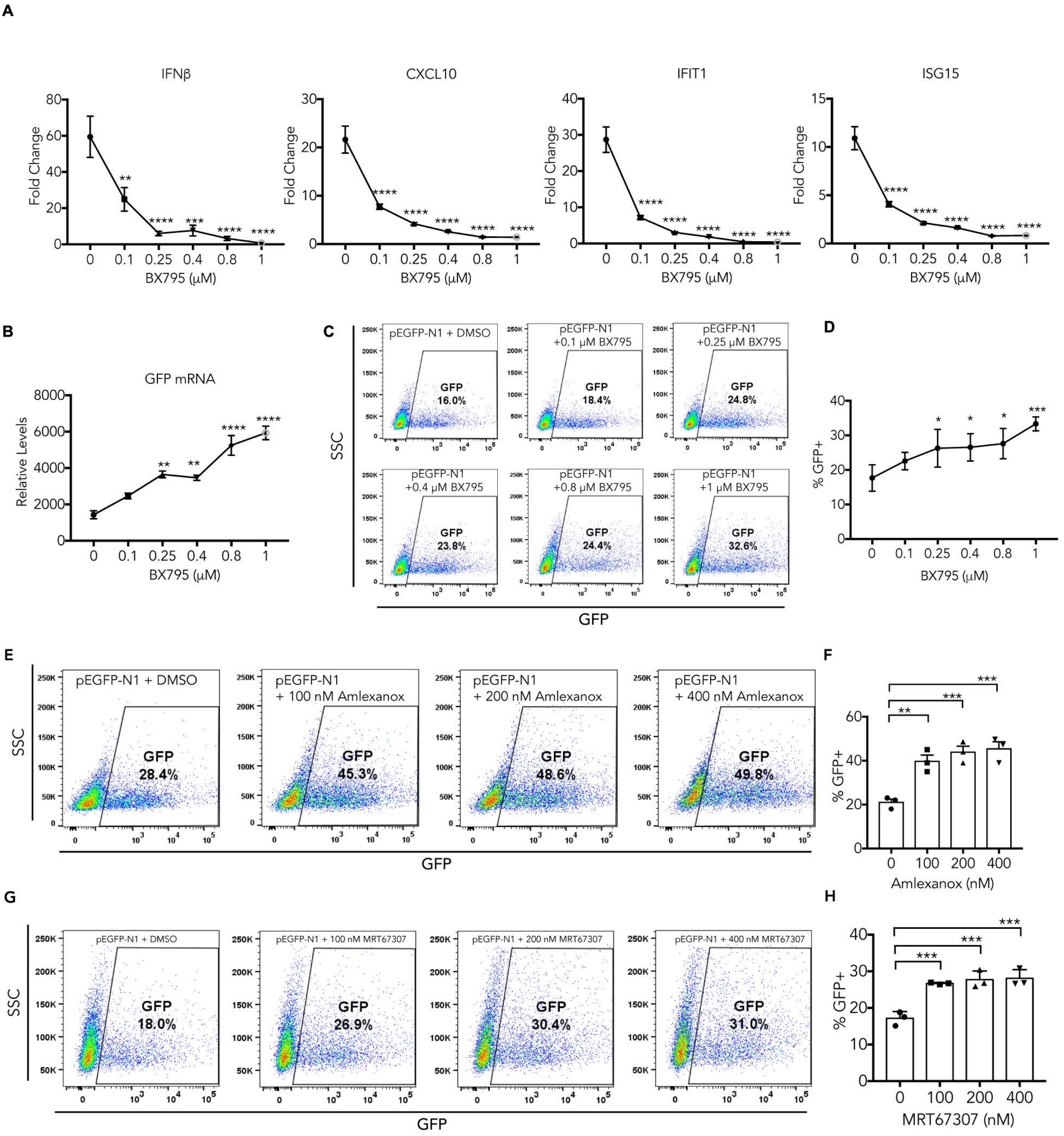

A
IFNβ
CXCL10
IFIT1
ISG15
Fold Change
BX795 (μM)
B
GFP mRNA
Relative Levels
BX795 (μM)
C
pEGFP-N1 + DMSO
pEGFP-N1 +0.1 μM BX795
pEGFP-N1 +0.25 μM BX795
pEGFP-N1 +0.4 μM BX795
pEGFP-N1 +0.8 μM BX795
pEGFP-N1 +1 μM BX795
GFP 16.0%
GFP 18.4%
GFP 24.8%
GFP 23.8%
GFP 24.4%
GFP 32.6%
SSC
GFP
D
% GFP+
BX795 (μM)
E
pEGFP-N1 + DMSO
pEGFP-N1 + 100 nM Amlexanox
pEGFP-N1 + 200 nM Amlexanox
pEGFP-N1 + 400 nM Amlexanox
GFP 28.4%
GFP 45.3%
GFP 48.6%
GFP 49.8%
SSC
GFP
F
% GFP+
Amlexanox (nM)
G
pEGFP-N1 + DMSO
pEGFP-N1 + 100 nM MRT67307
pEGFP-N1 + 200 nM MRT67307
pEGFP-N1 + 400 nM MRT67307
GFP 18.0%
GFP 26.9%
GFP 30.4%
GFP 31.0%
SSC
GFP
H
% GFP+
MRT67307 (nM)

Figure S3. Related to Figure 3. TBK1 inhibitors increase expression of transfected genes. A) L929 cells treated with increasing concentrations of BX795 were transfected with pEGFP-N1. After 8 hours, RNA levels of IFNβ, ISGs were quantified using RT-qPCR. **B)** The same as in A), except mRNA levels of GFP were quantified using RT-qPCR 24 hours after transfection. **C)** The same as A), except GFP positive cells were analyzed using FACS 24 hours after transfection. **D)** Quantification of data in C). In A), B), and D) Data represent mean ± SEM of 3 independent experiments. *p<0.05, **p<0.01, ***p<0.001, ****p<0.0001 (one-way ANOVA vs control, Dunnett's correction). **E)** L929 cells were treated with increasing concentrations of amlexanox and transfected with pEGFP-N1 plasmid. GFP expressing cells were analyzed by FACS 24 hours post transfection. **F)** Quantification and statistics of data in C). **G)** The same as E), except MRT67307 was used. **H)** Quantification and statistics of data in G). In F) and H), Data represent mean ± SEM of 3 independent experiments. **p<0.01, ***p<0.001 (one-way ANOVA vs control, Dunnett's correction).

Figure S4

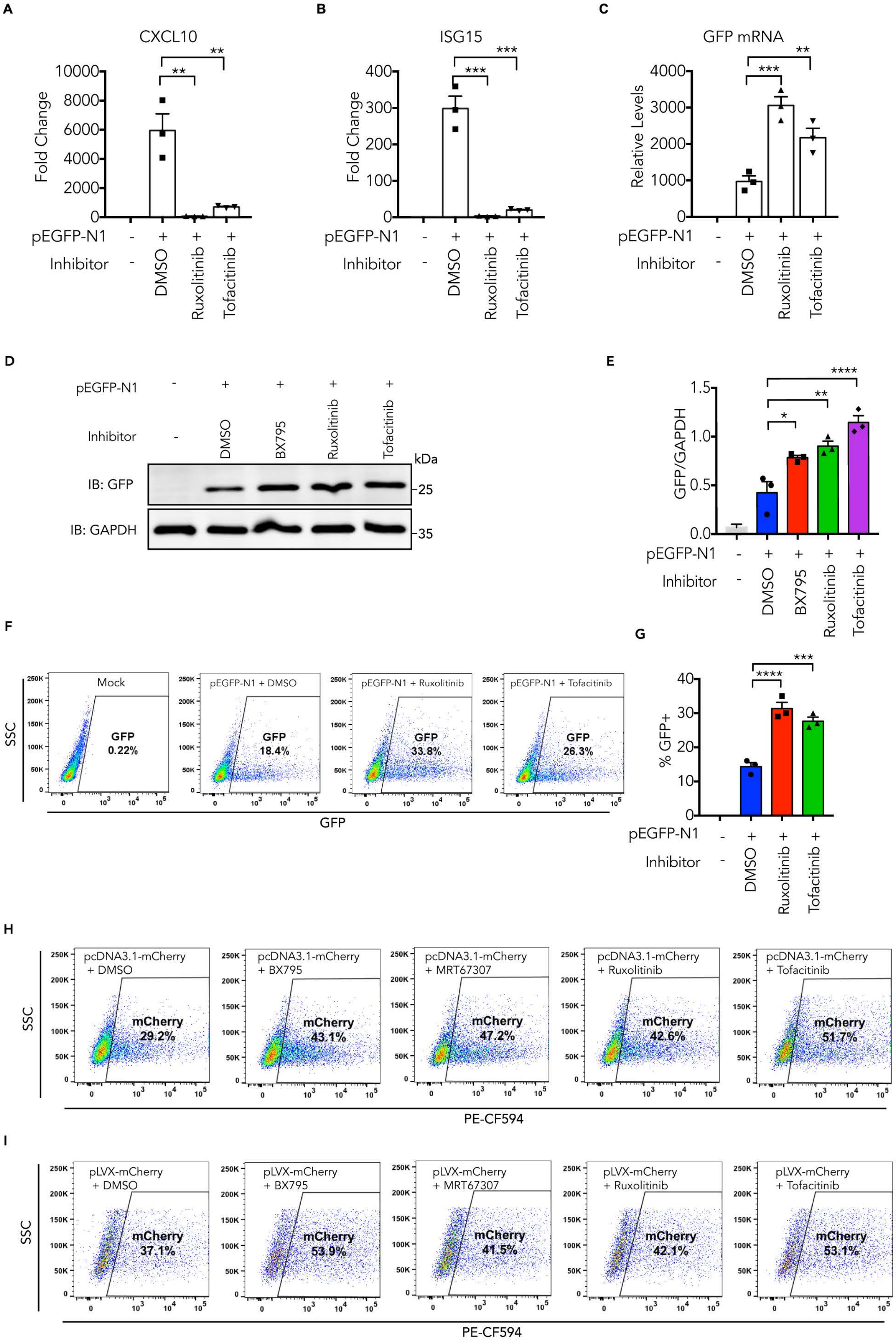

A
CXCL10
Fold Change
**
**
pEGFP-N1
Inhibitor
DMSO
Ruxolitinib
Tofacitinib
B
ISG15
Fold Change
***
***
C
GFP mRNA
Relative Levels
***
**
D
pEGFP-N1
Inhibitor
DMSO
BX795
Ruxolitinib
Tofacitinib
kDa
IB: GFP
25
IB: GAPDH
35
E
GFP/GAPDH
*
**
****
F
Mock
pEGFP-N1 + DMSO
pEGFP-N1 + Ruxolitinib
pEGFP-N1 + Tofacitinib
GFP 0.22%
GFP 18.4%
GFP 33.8%
GFP 26.3%
SSC
GFP
G
% GFP+
****
***
H
pcDNA3.1-mCherry + DMSO
pcDNA3.1-mCherry + BX795
pcDNA3.1-mCherry + MRT67307
pcDNA3.1-mCherry + Ruxolitinib
pcDNA3.1-mCherry + Tofacitinib
mCherry 29.2%
mCherry 43.1%
mCherry 47.2%
mCherry 42.6%
mCherry 51.7%
SSC
PE-CF594
I
pLVX-mCherry + DMSO
pLVX-mCherry + BX795
pLVX-mCherry + MRT67307
pLVX-mCherry + Ruxolitinib
pLVX-mCherry + Tofacitinib
mCherry 37.1%
mCherry 53.9%
mCherry 41.5%
mCherry 42.1%
mCherry 53.1%
SSC
PE-CF594

Figure S4. Related to Figure 3. JAK inhibitors increase expression of transfected genes. A, B, C) L929 cells were treated with JAK inhibitor Ruxolitinib (5 μM) or Tofacitinib (5 μM) and transfected with pEGFP-N1 plasmid. 8 hours later, RNA levels of CXCL10 (A), ISG15 (B), and GFP were measured by RT-qPCR. **D**) Same as in A-C), except protein levels of GFP was analyzed by Western blot. **E**) Quantification of intensity of GFP bands in (D). **F**) Same as in A-C), except GFP positive cells were analyzed by FACS. **G**) Quantification of GFP positive cells in F). In A), B), C), E), and G), Data represent mean ± SEM of >3 independent experiments. ***p<0.01, ***p<0.001, and ****p<0.0001* (one-way ANOVA vs DMSO control, Dunnett's correction). **H)** L929 cells were treated with indicated inhibitors and transfected with pcDNA3.1-mCherry for 24 hours. mCherry positive cells were analyzed by FACS. **I)** BJ-5ta cells were treated with indicated inhibitors and transfected with pLVX-mCherry for 24 hours. mCherry positive cells were analyzed by FACS.

Figure S5

A

PKR sgRNA TAAATATACCTTGGACGCC

WT GTATCCCCTGATGATGTGCAAAGAAATAAATATACCTTGGAC GCCAGGTAAGCGGAGTTTGTCTAA

GTATCCCCTGATGATGTGCAAAGAAATAAATATACCTTGGAC ·······CAGGTAAGCGGAGTTTGTCTAA (2 bp deletion)

L929 *Pkr*$^{-/-}$ GTATCCCCTGATGATGTGCAAAGAAATAAATATACCTTGGACCGCCAGGTAAGCGGAGTTTGTCTAA (1 bp insertion)

GTATCCCCTGATGATGTGCAAAGAAATAAATATACCTTGGACCTA······ GACGCCAGGTAAGCGGAGTTTGTCTAA (109 bp insertion)

B

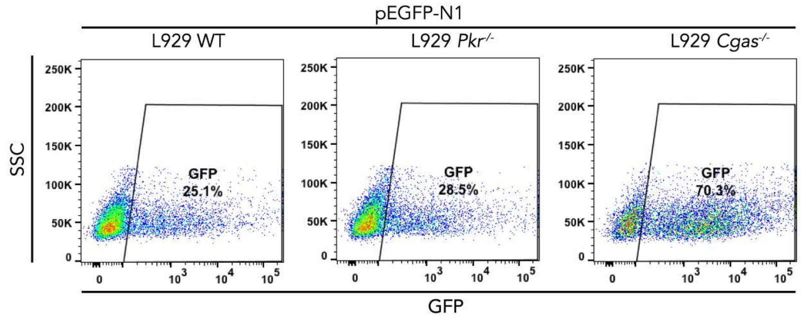

C

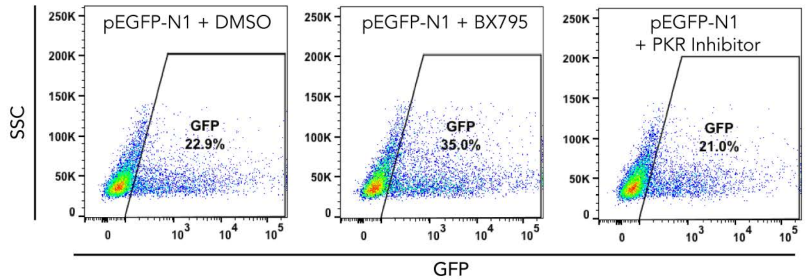

Figure S5. Related to Figure 4. PKR has no effect on transgene expression. A) Sequence analysis of *Pkr*$^{-/-}$ L929 cells. **B**) FACS analysis of GFP positive cells in wild-type, *Cgas*$^{-/-}$ or *Pkr*$^{-/-}$ L929 cells, 24 hours after transfection with pEGFP-N1 plasmid. **C**) L929 cells were treated with BX795 (0.5 μM) or PKR inhibitor (5 μM) and transfected with pEGFP-N1 plasmid. 24 hours later, GFP positive cells were analyzed by FACS.

Figure S6

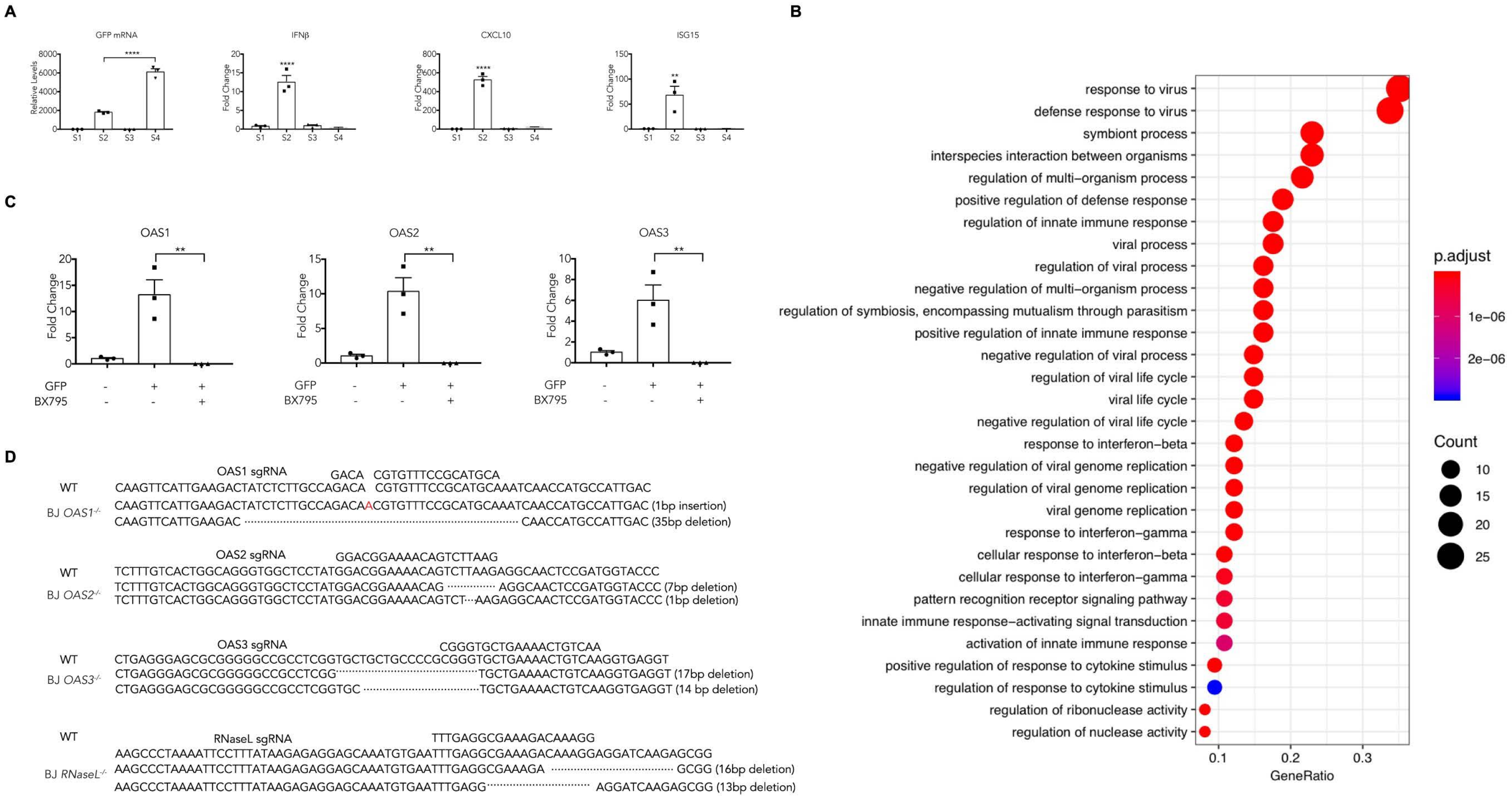
A
GFP mRNA
IFNβ
CXCL10
ISG15
Relative Levels
Fold Change
S1
S2
S3
S4
****
**
B
response to virus
defense response to virus
symbiont process
interspecies interaction between organisms
regulation of multi−organism process
positive regulation of defense response
regulation of innate immune response
viral process
regulation of viral process
negative regulation of multi−organism process
regulation of symbiosis, encompassing mutualism through parasitism
positive regulation of innate immune response
negative regulation of viral process
regulation of viral life cycle
viral life cycle
negative regulation of viral life cycle
response to interferon−beta
negative regulation of viral genome replication
regulation of viral genome replication
viral genome replication
response to interferon−gamma
cellular response to interferon−beta
cellular response to interferon−gamma
pattern recognition receptor signaling pathway
innate immune response−activating signal transduction
activation of innate immune response
positive regulation of response to cytokine stimulus
regulation of response to cytokine stimulus
regulation of ribonuclease activity
regulation of nuclease activity
GeneRatio
p.adjust
1e−06
2e−06
Count
10
15
20
25
C
OAS1
OAS2
OAS3
Fold Change
GFP
BX795
D
OAS1 sgRNA
GACA CGTGTTTCCGCATGCA
WT
CAAGTTCATTGAAGACTATCTCTTGCCAGACA CGTGTTTCCGCATGCAAATCAACCATGCCATTGAC
BJ OAS1-/-
CAAGTTCATTGAAGACTATCTCTTGCCAGACAACGTGTTTCCGCATGCAAATCAACCATGCCATTGAC (1bp insertion)
CAAGTTCATTGAAGAC ..... CAACCATGCCATTGAC (35bp deletion)
OAS2 sgRNA
GGACGGAAAACAGTCTTAAG
WT
TCTTTGTCACTGGCAGGGTGGCTCCTATGGACGGAAAACAGTCTTAAGAGGCAACTCCGATGGTACCC
BJ OAS2-/-
TCTTTGTCACTGGCAGGGTGGCTCCTATGGACGGAAAACAG ..... AGGCAACTCCGATGGTACCC (7bp deletion)
TCTTTGTCACTGGCAGGGTGGCTCCTATGGACGGAAAACAGTCT...AAGAGGCAACTCCGATGGTACCC (1bp deletion)
OAS3 sgRNA
CGGGTGCTGAAAACTGTCAA
WT
CTGAGGGAGCGCGGGGGCCGCCTCGGTGCTGCTGCCCCGCGGGTGCTGAAAACTGTCAAGGTGAGGT
BJ OAS3-/-
CTGAGGGAGCGCGGGGGCCGCCTCGG ..... TGCTGAAAACTGTCAAGGTGAGGT (17bp deletion)
CTGAGGGAGCGCGGGGGCCGCCTCGGTGC ..... TGCTGAAAACTGTCAAGGTGAGGT (14 bp deletion)
WT
RNaseL sgRNA
TTTGAGGCGAAAGACAAAGG
AAGCCCTAAAATTCCTTTATAAGAGAGGAGCAAATGTGAATTTGAGGCGAAAGACAAAGGAGGATCAAGAGCGG
BJ RNaseL-/-
AAGCCCTAAAATTCCTTTATAAGAGAGGAGCAAATGTGAATTTGAGGCGAAAGA ..... GCGG (16bp deletion)
AAGCCCTAAAATTCCTTTATAAGAGAGGAGCAAATGTGAATTTGAGG ..... AGGATCAAGAGCGG (13bp deletion)

Figure S6. Related to Figure 4. A) RT-qPCR measurement of mRNA levels of GFP, IFNβ, Cxcl10, and ISG15 in samples used for RNAseq in Fig 4A. **B)** Gene ontology enrichment analysis. **C)** BJ-5ta wild-type cells were treated with the TBK1 inhibitor BX795 and transfected with pEGFP-N1 plasmid. Induction of indicated OAS1, OAS2, OAS3 were measured by RT-qPCR. **D)** Sequence analysis of *OAS1*$^{-/-}$, *OAS2*$^{-/-}$, *OAS3*$^{-/-}$, and *RNaseL*$^{-/-}$ BJ-5ta cells. In A) and C), data represent mean ± SEM. ****p<0.01, ****p,0.0001* (one-way ANOVA with Dunnett's correction).

Figure S7

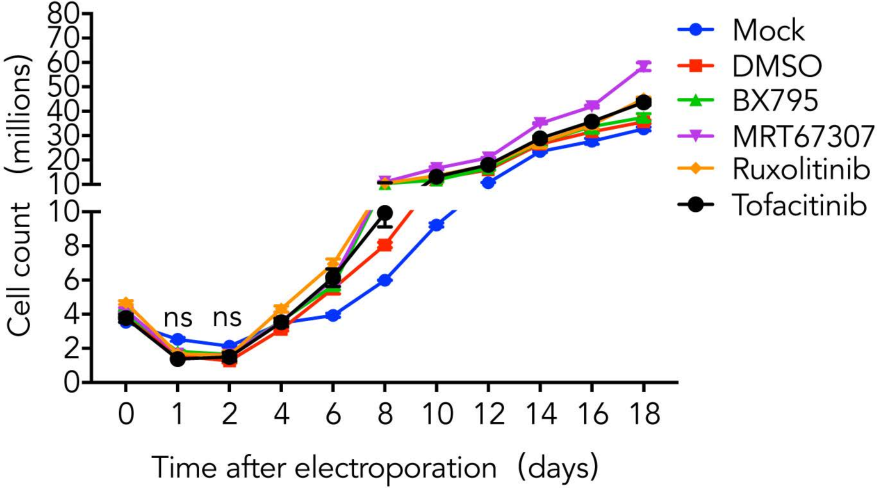

Cell count (millions)
80
70
60
50
40
30
20
10
10
8
6
4
2
0
ns
ns
0
1
2
4
6
8
10
12
14
16
18
Time after electroporation (days)
Mock
DMSO
BX795
MRT67307
Ruxolitinib
Tofacitinib

Figure S7. Related to Figure 6. Effect of inhibitors on cell growth of T cells after electroporation. CD3+ T cell proliferated from human peripheral blood were treated with indicated inhibitors (as in Fig. 6A) and electroporated with pEGFP-N1 plasmid. Live cells were counted on indicated days.

Figure S8

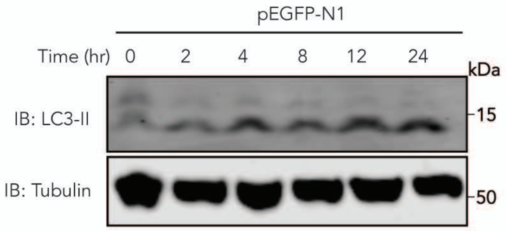

Figure S8. Related to Figure 4. Plasmid transfection induces autophagy. BJ-5ta cells were transfected with pEGFP-N1 with lipofectamine method for indicated time; lipidation of LC3 (LC3-II) was detected using Western blot.

Transparent Methods

Reagents and mice

Plasmid DNA pEGFP-N1 and pLVX-mCherry were obtained from Clontech Takara (Shiga, Japan). Plasmid pcDNA3.1-mCherry was obtained from Addgene (United States). The CMV promoter was cloned into pGL3-Enhancer (Promega, United States). This plasmid and plasmid pCMV - Renilla were used as vectors for measurement of luciferase activity. Purified mouse IFNβ was purchased from ProSpec-Tany Technogene (United States). LEAF™ Purified anti-mouse IFNAR-1 antibody was purchased from Biolegend (United States). The following chemicals were used and dissolved in dimethysulfoxide (DMSO): BX795 and MRT67307 (Selleck Chemicals, United States), Amlexanox (Invivogen, United States), Ruxolitinib and Tofacitinib (Apexbio Technology, United States), C-176 and C-178 (Biovision, United States).

Mouse anti-GFP 1E10H7 monoclonal antibody (1:1000; Proteintech), α-Tubulin Antibody (1:1000, Cell Signaling Technology), mouse anti-GAPDH 1E6D9 monoclonal antibody (1:1000; Proteintech), rabbit anti-OAS1 polyclonal antibody (1:500; Proteintech), rabbit anti-OAS2 H-180 polyclonal antibody (1:500; Santa Cruz), rabbit anti-OAS3 polyclonal antibody (1:1000; Proteintech), rabbit anti-RNaseL (1:500, Proteintech) were used to detect EGFP, GAPDH, Tubulin, OAS1, OAS2, OAS3, and RNaseL. cGAS Antibody (mouse specific) (1:1000, Cell Signaling Technology), MAVS Antibody (Rodent Specific) (1:1000, Cell Signaling Technology) and LC3B Antibody (1:1000, Cell Signaling Technology) was used to detect cGAS, MAVS, LC3B. IRDye 800CW goat anti-rabbit IgG and IRDye 800CW goat anti-mouse IgG were purchased from LI-COR Biosciences (United States).

Mice. *Cgas*$^{-/-}$, *Sting*$^{gt/gt}$, *Mavs*$^{-/-}$mice were from the Jackson laboratory. *Cgas*$^{-/-}$, *Sting*$^{gt/gt}$ were maintained on a C57BL/6J background. *Mavs*$^{-/-}$mice was maintained on a B6/129 background. C57BL/6 mice were purchased from SLAC Laboratory Animal LLC (Shanghai, China). Mice were bred and maintained under specific pathogen-free conditions in the Animal Center of Fujian Normal University. All research and animal care procedures were approved by the Animal Ethical and Welfare Committee of Fujian Normal University.

Cell lines, Primary Cells and Cell Culture

The cell lines including L929, BJ-5ta cells, Raw-ISG-luc cells; primary mouse embryonic fibroblasts (MEFs, mixed male and female), primary mouse lung fibroblasts (female), were

cultured in DMEM (Hyclone, United States) supplemented with 10% fetal bovine serum (FBS) (Gbico, United States) plus antibiotics at 37°C in an atmosphere of 5% (v/v) CO_2. Primary MEFs were prepared from day 13.5 embryos of C57BL/6 mice and seeded into 10 cm^2 cell culture dishes. In the following day, cells were split 1:3 and continued culture until reaching confluency for later use. To isolate primary lung fibroblasts, lung tissues from 6-8 weeks old female mouse were minced and digested in 5 ml of DMEM with 0.1% collagenase D (Roche, Switzerland) and 0.25% trypsin (Gbico, Thermo Fisher, United States) at 37°C for 45-60 min. After washing with DMEM, cells were seeded and cultured in a 10 cm^2 cell culture dish in 12 ml DMEM containing 10% FBS and antibiotics.

Cell transfection by Lipofectamine 2000 or electroporation

Cells were transfected with plasmid pEGFP-N1, pGL3-Enhancer, pCMV-Renilla , pcDNA3.1-mCherry or pLVX-mCherry using the Lipofectamine 2000 Reagent (Thermo Fisher, CA, United States) according to the manufacturer's instructions. Cells were electroporated using a Celetrix electroporator 1500A (Celetrix, Taizhou, China) as follows: 1×10^6 cultured T cells were suspended in an electroporation tube with 20 μl specific medium (provided by Celetrix) containing 3 μg plasmid DNA, and then cells were electroporated at 400V, 20 ms. PBMCs were electroporated at 840V, 20 ms. Cells were cultured for indicated periods after transfection or electroporation, and used for immunoblotting, flow cytometry, and RNA extraction.

RNA isolation and real-time PCR

Total RNA was extracted using Trizol reagent (Thermo Fisher, CA, United States) from cells according to the manufacturer's instruction. cDNA was generated using the PrimeScript™ II 1st Strand cDNA Synthesis Kit (Takara, Shiga, Japan). Quantitative real-time PCR (qPCR) was performed in triplicates using SYBR® Premix Ex Taq™ Mix (Takara, Shiga, Japan) and QuantStudio™ 6 Real-time PCR Instrument (Thermo Fisher, CA, United States). Relative expression levels were normalized to the levels of HPRT1 or RP13a. $2^{-\Delta\Delta Ct}$ method was used to calculate relative expression changes.

qPCR was performed using the following primers:

Gene	Forward Primer	Reverse Primer
mHPRT	CAGTCCCAGCGTCGTGATTAG	AAACACTTTTTCCAAATCCTCGG
mIFNβ	TCCGAGCAGAGATCTTCAGGAA	TGCAACCACCACTCATTCTGAG
mIFIT1	TGGCCGTTTCCTACAGTT	TCCTCCAAGCAAAGGACTTC

mISG15	CTGAAGAAGCAGATTGCCCAGAAG	CGCTGCAGTTCTGTACCACTAGC
mCXCL10	GCCGTCATTTTCTGCCTCA	CGTCCTTGCGAGAGGGATC
EGFP	AGTCCGCCCTGAGCAAAGA	TCCAGCAGGACCATGTGATC
hRP13a	CGAGGTTGGCTGGAAGTACC	CTTCTCGGCCTGTTTCCGTAG
hOAS1	CAAGCTCAAGAGCCTCATCC	TGGGCTGTGTTGAAATGTGT
hOAS2	AGAAGCCAACGTGACATCCT	CAAGGGACTTCTGGATCTCG
hOAS3	GTCAAACCCAAGCCACAAGT	GGGCGAATGTTCACAAAGTT
hCXCL10	TGGCATTCAAGGAGTACCTC	TTGTAGCAATGATCTCAACACG
hISG15	GAGCATCCTGGTGAGGAATAAC	CGCTCACTTGCTGCTTCA

Western blot analysis

After drug treatments for indicated periods, cells were transfected with plasmid pEGFP-N1. At the various time points post-transfection, the medium was removed and the cells were collected in lysis buffer [50 mM Tris-Cl, PH 7.5, 100 mM NaCl, 1 mM EDTA, 0.5 mM EGTA, 10% Glycerol, 0.5% NP40] containing protease and phosphatase inhibitors (Thermo Fisher, CA, United States) and incubated at 4°C for 10 min. Cell lysates were centrifuged at 14,000 rpm at 4°C for 5 min, and the supernatants were collected. Total protein concentrations were determined by BCA protein assay kit (Thermo Fisher, CA, United States). Equal amounts of proteins were separated by sodium dodecyl sulfate-polyacrylamide gel electrophoresis (SDS-PAGE) and were transferred onto a polyvinylidene difluoride membrane (Thermo Fisher, CA, United States). The membranes were blocked with 5% BSA in Tris-buffered saline for 1 hour at room temperature, followed by incubation with the primary antibody. After incubation with IRDye 800CW goat anti-rabbit IgG or goat anti-mouse IgG second antibody, the reactive bands were detected on the Odessey CLx Imaging systems (LI-COR, Biosciences, United States). Glyceraldehyde-3-phosphate dehydrogenase (GAPDH) or tubulin were used as loading control. The protein expression levels were determined by fluorescent intensity as analyzed with NIH Image J software.

Culture and proliferation of human T cells: Human blood samples were obtained from Fujian Normal University Clinics with the consent agreements with the healthy human volunteers, which was approved by the FJNU ethnic committee. PBMC was isolated by Ficoll gradient (GE life sciences, United States) from healthy blood donors and cultured in X-VIVO™ 15 Serum-free Hematopoietic Cell Medium (Lonza, United States) and activated with 1000 U/ml human IL-2 (T&L Biological Technology, China) and 2μg/ml human CD3/CD28 antibodies (T&L Biological Technology, China) for 6 days. Afterwards cells were fed with fresh X-VIVO™ 15 media and 500

U/ml human IL-2 every 2 days. Totally 10 days later, cells were counted and their phenotypes (CD45+CD3) were confirmed with FACS.

Flow Cytometry Analysis

EGFP-expressing cells were determined quantitatively by an FACS Aria III cell sorter or BD FACSymphony^{TM} system (Becton Dickinson, CA, United States). All samples were suspended in PBS and filtered to 70 μm nylon mesh filter just prior to analysis. Voltage and gain settings were 300 and 1.00 in log mode for FL1 (green fluorescence) readings, respectively. For surface staining, samples were incubated with antibodies in staining buffer for 30 min at 4°C. Antibodies used for staining were: Fixable viability stain 450, CD3-APC, CD69-APC Cy7, CD62L-PE (BD Biosciences). Typically, 10,000 cells were analyzed per sample. For analysis of EGFP expression in PBMC, cells transfected for 24 hours were first gated on FSC/SSC to identify lymphocytes population, which is further gated for live cells using FVS-BV450 followed by GFP gating to evaluate population of transfected cells. Cell were sequentially gated for CD3+ → CD69-CD62L+ → GFP+ for analysis of EGFP expression in resting T cells. All cell acquisition was performed using a BD FACSymphony^{TM} system (Becton Dickinson, CA, United States). Data acquisition and analysis were performed using the flowjo software from BD (Becton Dickinson, CA, United States).

RNA-seq Analysis

Total RNA was isolated as described above, and cDNA library construction and sequencing were performed by BGI Genomics (BGI-SHENZHEN, China) using BGISEQ-500 platform. High-quality reads were aligned to the Mus_musculus reference genome (Mus musculus GRCm38.p5) using Bowtie2 (version:2.2.5, parameters: -q --phred64 --sensitive --dpad 0 --gbar 99999999 --mp 1,1 --np 1 --score-min L,0,-0.1 -p 16 -k 200). The expression levels for each of the genes were normalized to fragments per kilobase of exon model per million mapped reads (FPKM) using RNA-seq by Expectation Maximization (RSEM) (version:1.2.12, default parameters). For the identification of differentially expressed genes (DEGs), we used PossionDis, which is developed by BGI Genomics, implementing the passion distribution method. A false discovery rate (FDR) cutoff of 0.001 and a log-fold change (LogFC) threshold of 2 were implemented to filter the significantly different genes. The genes with logFC ⩾ 2 and logFC ⩽ -2 with FDR ⩽ 0.001 between two treatment conditions were determined to be up-regulated and down-regulated genes. For Gene Ontology (GO) analysis, clusterProfiler (version 3.6.0) R package (version 3.4.4)

was used to test the statistical enrichment of marker genes in KEGG pathways; GO terms with corrected P value less than 0.05 were considered significantly enriched by marker genes.

Generation of Gene Knockout Cells using CRISPR/Cas9 technology

To knock out the targeted genes, the specific sgRNAs were designed using the website http://crispr.mit.edu/ and were inserted into pPX459 as described previously (Cong et al., 2013; Mali et al., 2013). 2 μg of the recombinant plasmids containing the specific target sgRNAs (as indicated in the corresponding figures) were used for transfection or electroporation as described above. Forty-eight hours post transfection or electroporation, the cells were cultured in the presence of puromycin (8 μg/ml for L929 cells or 0.5 μg/ml for BJ-5ta cells). After 3 days, the medium was replaced with fresh medium without puromycin and culture were continued until cells reached confluency. The cells were then trypsinized and cloned by limited dilution. Single-cell clones were selected for conducting further experiments.

Immunoprecipitation

BJ-5ta cells or L929 cells were transfected with pCMV-RNaseL-Flag for 24 hours, then gently lysed with lysis buffer [50 mM Tris-Cl, PH 7.5, 100 mM NaCl, 1 mM EDTA, 0.5 mM EGTA, 10% Glycerol, 0.5% NP40] containing protease and phosphatase inhibitors (Thermo Fisher, CA, United States) and incubated at 4°C for 10 min. Cell lysates were centrifuged at 14,000 rpm at 4°C for 5 min, and the supernatants were collected. The supernatant was incubated with 10 μL Anti-FLAG® M2 Magnetic Beads (Millipore, Germany) at 4°C for 3 hrs. The immunoprecipitated complexes were washed five times and eluted with 30 μL lysis buffer supplemented with protease and phosphatase inhibitors (Thermo Fisher, CA, United States). 10 μL elution were heated at 95°C for 10 min in 1 X SDS/sample buffer supplemented with 5% β-mercaptoethanol and used for western blot analysis. The elution were measured for RNaseL activity immediately.

Assay of RNaseL activity

The assay of RnaseL activity was performed basically as previously described (Townsend et al., 2008). Briefly, a labeled RNA probe was derived from a 36-nt intergenic sequence of respiratory syncytial virus (RSV) (UUAUCAAAUUCUUAUUUGCCCCAUUUUUUUGGUUUA) with a fluorophore (6FAM) at the 5' terminus. The elution of RNaseL protein was incubated with 100 nM RNA probe at room temperature in the dark for 1.5 hrs. The reactions products were examined

by TBE electrophoresis with 5 × Novex Hi-Density TBE Sample Buffer (Thermo Fisher, United States).

Statistical analysis

All statistical analysis was performed using GraphPad Prism software (GraphPad, La Jolla, CA, United States). Details were described in figure legends.

Supplemental References

Cong, L., Ran, F.A., Cox, D., Lin, S., Barretto, R., Habib, N., Hsu, P.D., Wu, X., Jiang, W., Marraffini, L.A., *et al.* (2013). Multiplex genome engineering using CRISPR/Cas systems. Science *339*, 819-823.

Mali, P., Yang, L., Esvelt, K.M., Aach, J., Guell, M., DiCarlo, J.E., Norville, J.E., and Church, G.M. (2013). RNA-guided human genome engineering via Cas9. Science *339*, 823-826.

Townsend, H.L., Jha, B.K., Han, J.Q., Maluf, N.K., Silverman, R.H., and Barton, D.J. (2008). A viral RNA competitively inhibits the antiviral endoribonuclease domain of RNase L. RNA *14*, 1026-1036.
